# Antagonistic functions of CTL1 and SUH1 mediate cell wall assembly in *Arabidopsis*

**DOI:** 10.1002/pld3.580

**Published:** 2024-03-23

**Authors:** Nguyen Thi Thuy, Hyun‐Jung Kim, Suk‐Whan Hong

**Affiliations:** ^1^ Department of the Integrative Food, Bioscience, and Biotechnology, College of Agriculture and Life Sciences Chonnam National University Gwangju Korea

**Keywords:** cell wall, chitinase‐like protein, Domain of Unknown Function 266 (DUF266), genetic suppression, glycosyltransferase, Golgi complex

## Abstract

Plant genomes contain numerous genes encoding chitinase‐like (CTL) proteins, which have a similar protein structure to chitinase belonging to the glycoside hydrolase (GH) family but lack the chitinolytic activity to cleave the *β*‐1,4‐glycosidic bond in chitins, polymers of *N*‐acetylglucosamine. *CTL1* mutations found in rice and *Arabidopsis* have caused pleiotropic developmental defects, including altered cell wall composition and decreased abiotic stress tolerance, likely due to reduced cellulose content. In this study, we identified *suppressor of hot2 1* (*suh1*) as a genetic suppressor of the *ctl1* ^*hot2‐1*^ mutation in *Arabidopsis*. The mutation in *SUH1* restored almost all examined *ctl1* ^*hot2‐1*^ defects to nearly wild‐type levels or at least partially. *SUH1* encodes a Golgi‐located type II membrane protein with glycosyltransferase (GT) activity, and its mutations lead to a reduction in cellulose content and hypersensitivity to cellulose biosynthesis inhibitors, although to a lesser extent than *ctl1* ^*hot2‐1*^ mutation. The *SUH1* promoter fused with the GUS reporter gene exhibited GUS activity in interfascicular fibers and xylem in stems; meanwhile, the *ctl1* ^*hot2‐1*^ mutation significantly increased this activity. Our findings provide genetic and molecular evidence that the antagonistic activities of CTL1 and SUH1 play an essential role in assembling the cell wall in *Arabidopsis*.

## 1 INTRODUCTION

Chitinases (EC 3.2.1.14) comprise a subfamily of glycoside hydrolases (GHs) that catalyze the hydrolysis of β‐1,4 glycosidic bonds in amino polysaccharides such as chitin and chitooligosaccharides (Kesari et al., 2015). They are primarily grouped into six classes, I to VI. Classes III and V belong to the GH18 family, which exhibits a TIM (triosephosphate isomerase) fold consisting of a β/α barrel structure (Funkhouser & Aronson, 2007). The other classes belong to the GH19 chitinases, which are composed of an α‐helical structure in the catalytic region (Ohnuma et al., 2012). In addition to the well‐known role of chitinases in plant defense mechanisms against fungal pathogens, there have been several reports of chitinase‐like proteins (CTLs) participating in cell wall synthesis in *Arabidopsis* and rice, ultimately affecting their growth and development (Hauser et al., 1995; Hermans et al., 2010; Wu et al., 2012; Zhong et al., 2002). CTLs, which have a catalytic region composed of an α‐helical structure, are structurally characterized by the absence of a hevein domain, known as the chitin‐binding domain, and the chitinase activity motif (H‐E‐E‐T). Hence, these features allow them to be further categorized as class II, which belongs to the GH19 family (Grover, 2012). Although no chitinolytic activity was detected (Kwon et al., 2007; Wu et al., 2012), CTLs in *Arabidopsis* and Caribbean pine were reported to bind to glucan chains and arabinogalactan, respectively (Domon et al., 2000; Sánchez‐Rodríguez et al., 2012). These findings suggest that these interactions affect the cell wall assembly, which is composed mainly of complex polysaccharides.

Plant cell walls (CWs) can be classified into primary and secondary CWs, which differ in composition and function (Houston et al., 2016; Rui & Dinneny, 2019). The primary CW is relatively thin and flexible, consisting of cellulose microfibrils embedded in a matrix of various polysaccharides such as hemicelluloses, pectins, and glycoproteins. It determines cell shape and controls cell expansion during growth, contributes to the response to environmental stimuli, and allows for cell–cell communication. The secondary CW is thicker and more rigid than the primary CW. It is deposited on the inner side of the primary CW after cell expansion has ceased. The secondary CW mainly comprises cellulose microfibrils, hemicelluloses, and lignin, which is a polyphenolic compound that provides rigidity and waterproofing properties to the secondary CW. Consequently, depositing secondary CWs in interfascicular cells and xylem elements is crucial for providing structural support and facilitating water transport.

Various mutations affecting cell wall synthesis have resulted in abnormal growth and development, demonstrating the biological importance of CWs in plants. Cellulose is synthesized at the plasma membrane by a large membrane‐bound protein complex. The catalytic core comprises three cellulase synthase (CESA) types in the primary and secondary CWs (Endler & Persson, 2011). In *Arabidopsis*, the CESA complex, which contains CESA1 (RADIAL SWELLING1 [RSW1]), CESA3 (ISOXABEN RESISTANT1 [IXR1]), and CESA6 (PROCUSTE1[PRC1]) subunits, is mainly responsible for cellulose synthesis in the primary CW. Insertion of T‐DNA into *CESA1* and *CESA3* leads to lethal male gametophytes (Persson, Paredez, et al., 2007). However, a null mutation in *CESA6* (*cesa6* ^*prc1‐1*^) exhibits mild phenotypes, such as reduced root and etiolated hypocotyl growth (Fagard et al., 2000). Genetic evidence shows that mutations in *CESA4* (*IRREGULAR XYLEM5* [*IRX5*]), *CESA7* (*IRX3*), and *CESA8* (*IRX1*), which are involved in cellulose synthesis in the secondary CW, cause a collapse in xylem development (Chen et al., 2005; Taylor et al., 1999, 2003). In addition, the small stature of many mutants defective in noncellulose polysaccharides such as pectin (Bouton et al., 2002; Liwanag et al., 2012) and hemicellulose (Xiao et al., 2016) provides genetic evidence of the physiological roles of the cell wall. Mutations in the *Arabidopsis QUASIMODO1* (*QUA1*), which encodes a protein with amino acid sequence similarity to α‐1,4‐D‐galacturonosyltransferase that belongs to the glycosyltransferase 8 (GT8) family, cause a 25% reduction in pectin, leading to dwarfism and reduced cell adhesion (Bouton et al., 2002). The *Arabidopsis* mutations in *IRX8* (encoding a protein similar to α‐1,4‐D‐galacturonosyltransferase, belonging to the GT8 family) exhibit a significant decrease in hemicellulose, such as homogalacturonan and xylan and display dwarfism and a reduction in secondary cell wall thickness (Persson, Caffall, et al., 2007). Moreover, rice *brittle culm* (*bc*) mutants with reduced mechanical internode strength also demonstrate the essential role cell walls perform in growth and development (Zhang & Zhou, 2011).

Despite numerous reports that CTLs are involved in cell wall assembly in *Arabidopsis* and rice, their lack of enzymatic activity and the structural complexity of their potential substrates—polysaccharides—further complicate the understanding of their functions. Therefore, to overcome these difficulties, we employed a genetic approach to further comprehend their function in cell wall assembly by isolating suppressor mutations that alleviate the defects caused by *CTL1* mutations in *Arabidopsis*. Here, we report that the *suppressor of hot2 1* (*SUH1*) gene encodes a Golgi‐localized type II membrane protein with glycosyltransferase activity, which is also potentially involved in glycan synthesis. Our findings provide genetic evidence that the interaction between CTL1 and SUH1, which have opposing enzymatic activities in glycan synthesis, plays a vital role in cell wall assembly in *Arabidopsis*.

## 2 MATERIALS AND METHODS

### 2.1 Plant growth conditions

Seeds were surface‐sterilized and grown on half‐strength (1/2) Murashige and Skoog (MS) media supplemented with 1% (w/v) sugar and .8% (w/v) agar, adjusted to pH 5.8 with KOH, either in total darkness or under long‐day conditions (16 h light/8 h dark, 22°C/18°C cycle under light density of 120 μmol m^−2^ s^−1^) in a growth chamber. Seedlings (light grown on 1/2 MS media) were transferred to the soil at 10 days old and grown under long‐day conditions in a growth chamber (light density of 120 μmol m^−2^ s^−1^). Unless stated otherwise, all mutants and transgenic lines used in this study were obtained from the *Arabidopsis* Columbia‐0 ecotype (Col‐0). We received the *Arabidopsis thaliana* SAIL_912_D02 T‐DNA insertion line (*suh1‐4*) with T‐DNA inserted in the first intron of *At5g14550* from the *Arabidopsis* Information Resource (TAIR). Genomic DNA was extracted from individual plants. To confirm T‐DNA insertion, PCR amplification was performed using gene‐specific primers and the T‐DNA left border primer LBb1 (Table S2). The precise position of the T‐DNA insertion was determined by sequencing the PCR products using the T‐DNA left border primer LBb1.

### 2.2 EMS mutagenesis and isolation of the suppressor of *ctl1* ^*hot2‐1*^ mutation

Approximately 10,000 *ctl1* ^*hot2‐1*^ seeds (M_1_ seeds) were imbibed overnight in water at 4°C before incubation in 50 ml of .25% ethyl methane sulfonate (EMS) for 12 h. Subsequently, the EMS was washed several times with water. The M_2_ seeds were independently harvested from 100 trays, with each containing about 100 M_1_ plants. Since the dark‐grown *ctl1* ^*hot2‐1*^ seedlings display a short hypocotyl, about 120,000 M_2_ seeds were grown in the dark to identify three independent seedlings with a long hypocotyl. Then, it was confirmed that these three suppressors grown in the dark also showed a long hypocotyl in the successive M_3_ generation, and they were designated as *suh* (suppressor of *hot2*) mutants. To determine the inheritance characteristics of the suppressor mutation, three *suh* mutant plants were crossed with the parental *ctl1* ^*hot2‐1*^ plants, and all pairwise combinations of the three *suh* mutants were crossed to conduct allelism tests.

### 2.3 Map‐based cloning of *SUH1* gene

To perform map‐based cloning of the *suh1* mutation, we looked for a *ctl1* mutant allele in the *Landsberg erecta* (Ler) ecotype by conducting a genetic screening of EMS‐mutagenized Ler seeds. Complementation testing and sequencing analysis revealed that a new *ctl1* allele (Ler) harbors the same mutation as the *ctl1* ^*hot2‐1*^ (G881A).

To generate a mapping population, *ctl1* ^*hot2‐1*^ *suh1‐1* mutant (Col‐0) was crossed with Ler‐derived *ctl1* plants named *ctl1* ^*hot2‐3*^. The resulting F_1_ plants were self‐pollinated, and DNA was extracted from individual F_2_ plants displaying the wild‐type phenotype of seedlings grown in the dark. PCR was performed using simple sequence length polymorphism (SSLP) markers (Bell & Ecker, 1994). The recombination frequency between the *SUH1* locus and the SSLP makers was determined to identify the position of the *SUH1* locus.

### 2.4 Semiquantitative RT‐PCR and cDNA synthesis

Total RNA was extracted from stems of *Arabidopsis* and rice using the Ribospin™ kit from GeneAll (https://geneall.com/). For RT‐PCR, 2–5 μg of total RNA was reverse transcribed using a Superscript First‐Strand Synthesis system (Invitrogen, Carlsbad, CA, USA). The quantitative RT‐PCR was performed using the *suh1‐4* RT primers (Table S2) and *Arabidopsis actin2* as an endogenous reference. PCR products were visualized by agarose gel electrophoresis using EtBr staining.

First‐strand cDNA was synthesized from 5 μg of total RNA using the PrimeScript™II cDNA Synthesis kit from Takara (https://takara.com/). Full‐length cDNAs of *SUH1* and *BC10* were amplified using the Taq LA DNA polymerase PCR kit from Takara (https://takara.com/) and cDNA‐specific primers (Table S2). The resulting fragments were cloned into the pGEM‐T easy vector and sequenced using an ABI 3730 automated sequencer (Applied Biosystems).

### 2.5 Construction of various vectors and plant transformation

The promoter fragment, located between −1060 and −1 in the *SUH1* gene, was amplified (the A site in the ATG translation start codon was designated +1). The *pSUH1::GUS* construct was generated by replacing the 35S promoter in the pBI121 binary vector with a fragment containing the *SUH1* promoter. To develop the constructs used in the complementation tests, the full‐length cDNAs of *SUH1* and *BC10* were placed under the control of the *SUH1* promoter sequence (1060 bp) in the pBI121 binary vector. The resulting binary vectors (*pSUH1::SUH1* and *pSUH1::OsBC10*) were introduced into the *Agrobacterium tumefaciens* strain GV3101 for *Arabidopsis* transformation using the floral dip method (Clough & Bent, 1998). Seedlings from the T_1_ generation were selected on half‐strength MS medium containing 30 μg/ml kanamycin. T_3_ homozygous progenies were isolated for each transgene, and three independent lines were selected for each construct for further examination.

### 2.6 Cell wall composition assay and histology

The Updegraff assay was applied to determine the cellulose content in the inflorescence stems (the bottom 5 cm of the stem) from 6‐week‐old wild‐type and mutant plants (Kumar & Turner, 2015). Samples were incubated in 70% ethanol at 70°C for 1 h, washed with 100% acetone, and dried overnight. After adding the acetic nitric agent (8:1:2, acetic acid:nitric acid:water), the samples were placed in a bath with boiling water for 30 min, precipitated by centrifugation at 14,000 rpm for 15 min, and incubated in 67% sulfuric acid in boiling water for 5 min. Finally, the anthrone–sulfuric acid colourimetric assay was performed to analyze the samples. The absorbance was measured at 620 nm using a spectrometer (UV‐1600, Shimadzu). The cellulose contents were expressed as the percentage of cellulose in the cell wall composition, with glucose standards.

Hand‐cut sections of inflorescence stems were incubated in 1% (w/v) phloroglucinol in 18% HCl, .01% (w/w) ruthenium red or 2 mg/ml β‐glycosyl Yariv agent in .1‐M NaCl to stain the lignin, pectin, and arabinogalactan proteins, respectively. After staining, the samples were washed with deionized water and observed using a Carl Zeiss AX10 microscope (Zeiss Corp, Germany).

To determine the shapes of the pith cells, the inflorescence stems of 6‐week‐old wild‐type and mutant plants were fixed in 2.5% glutaraldehyde at 4°C overnight. Tissues were gradually dehydrated in ethanol and embedded in Historesin (Leica Microsystems, France) according to the manufacturer's instructions. Transverse sections (5‐μm‐thick) were prepared using an ultramicrotome (LEICA, Germany), stained with 1% toluidine blue, and examined using a Carl Zeiss AX10 microscope (Zeiss Corp, Germany).

### 2.7 Treatment with cellulose biosynthesis inhibitors and abiotic stress

To determine their sensitivity to cellulose biosynthesis inhibitors (CBIs), sterilized *Arabidopsis* seeds were grown vertically on a half‐strength MS medium supplemented with the indicated concentrations of 2,6‐dichlorobenzonitrile (DCB) or isoxaben (ISX) in .5% dimethyl sulfoxide (DMSO). An equal volume of DMSO was used as a control. The primary root growth was measured after10 days.

To assess thermotolerance, seedlings were grown vertically in the dark for 2.5 days, subjected to heat stress at 45°C for 2 h, and further grown in the dark for 2.5 days. To apply salinity stress, 3‐day‐old seedlings grown vertically on a half‐strength MS medium were transferred to a medium with or without the indicated concentrations of NaCl. After 7 days of growth under long‐day conditions (16 h light/8 h dark), the primary root lengths of wild‐type and mutant plants were measured.

### 2.8 Histochemical assay for GUS activity

Histochemical analysis of β‐glucuronidase (GUS) activity was conducted as described previously (Jefferson et al., 1987). GUS enzyme activity in transgenic plants was determined by staining with 1 mg/ml X‐Gluc (Duchefa, the Netherlands) as the substrate. The GUS‐stained tissues in this report represent the typical results obtained in three independent transgenic lines.

### 2.9 Assay of C2GnT enzyme activity

The C2GnT assay was performed as described previously (Bierhuizen & Fukuda, 1992). To this end, *SUH1* and *BC10* were fused in‐frame with *EGFP* using the pEGFP‐N1 vector. The plasmids were transiently transfected into Chinese hamster ovary (CHO) cells. After 48 h, the cells were washed with phosphate‐buffered saline, and suspended in lysis buffer (10‐mM TrisCl at pH 8.0, .1‐mM EDTA, 5‐mM DTT, .9% NaCl, and 1% Triton X‐100). The cell lysate was centrifuged at 1000 *g* at 4°C for 10 min. Then, the supernatant was stored in aliquots of 250 μl at −80°C until further use. Protein concentration was determined using a Bio‐Rad protein assay with bovine serum albumin (BSA) as the standard. As the accepter, Gal*β*1 → 3GalNAc*α*1 → p‐nitrophenyl (Sigma) was employed for the C2GnT assay. Nontransfected and empty pEGFP‐N1‐transfected CHO cells were used as the negative control group. The reaction mixtures contained 50‐mM HEPES‐NaOH at pH 7.0, 1‐mM UDP‐[glucosamine‐U‐^14^C] GlcNAc (3.7 kBq, Amersham Pharmacia Biotech), 1‐mM acceptor oligosaccharide, .1‐M GlcNAc, and 5‐mM DTT. After incubating at 37°C for 3 h, the reaction products were adjusted to .25‐M ammonium formate at pH 4.0 and applied to a C18 reverse phase column (Alltech Associates Inc, IL, United States). After washing the column using the same solution, the product was eluted using 70% methanol. The radioactivity was measured using scintillation counting.

### 2.10 Tobacco transient expression and confocal microscopic analysis of SUH1–GFP fusion proteins

To investigate its subcellular location, *SUH1* was fused in‐frame with the GFP‐encoding sequence of the pCAMBIA1300 vector. The ER marker mCherry‐HDEL and the Golgi marker MAN49‐mCherry were obtained from the Daegu Gyeongbuk Institute of Science and Technology (DGST). Transient transformations of *Nicotiana benthamiana* with *A. tumefaciens* strain (GV3101) containing the *SUH1‐GFP* construct and organelle markers were conducted. The *Agrobacterium* cells were incubated in an infiltration buffer (10‐mM MgCl_2_, 10‐mM MES at pH 5.9, and 150‐μM acetosyringone) at room temperature for 3 h. Before infiltration, the bacterial suspension was mixed with an equal volume of a bacterial suspension harboring pBin61‐P19 to co‐introduce the RNA‐silencing suppressor gene into the cells. Next, the mixture of *Agrobacterium* suspensions was infiltrated into the abaxial side of the second, third, and fourth leaves of 6‐week‐old *N. benthamiana* plants using a 5‐ml syringe without a needle and grown in a growth chamber at 25°C under long‐day conditions (16 h light/8 h dark). The agroinfiltrated plants were placed back into the same growth chamber, and their fluorescence was examined using a Carl Zeiss LSM 710 confocal scanning microscope.

## 3 RESULTS

### 3.1 *SUH1* mutations restore the growth retardation of *ctl1* ^*hot2‐1*^ seedlings grown in the dark

**FIGURE 1:**
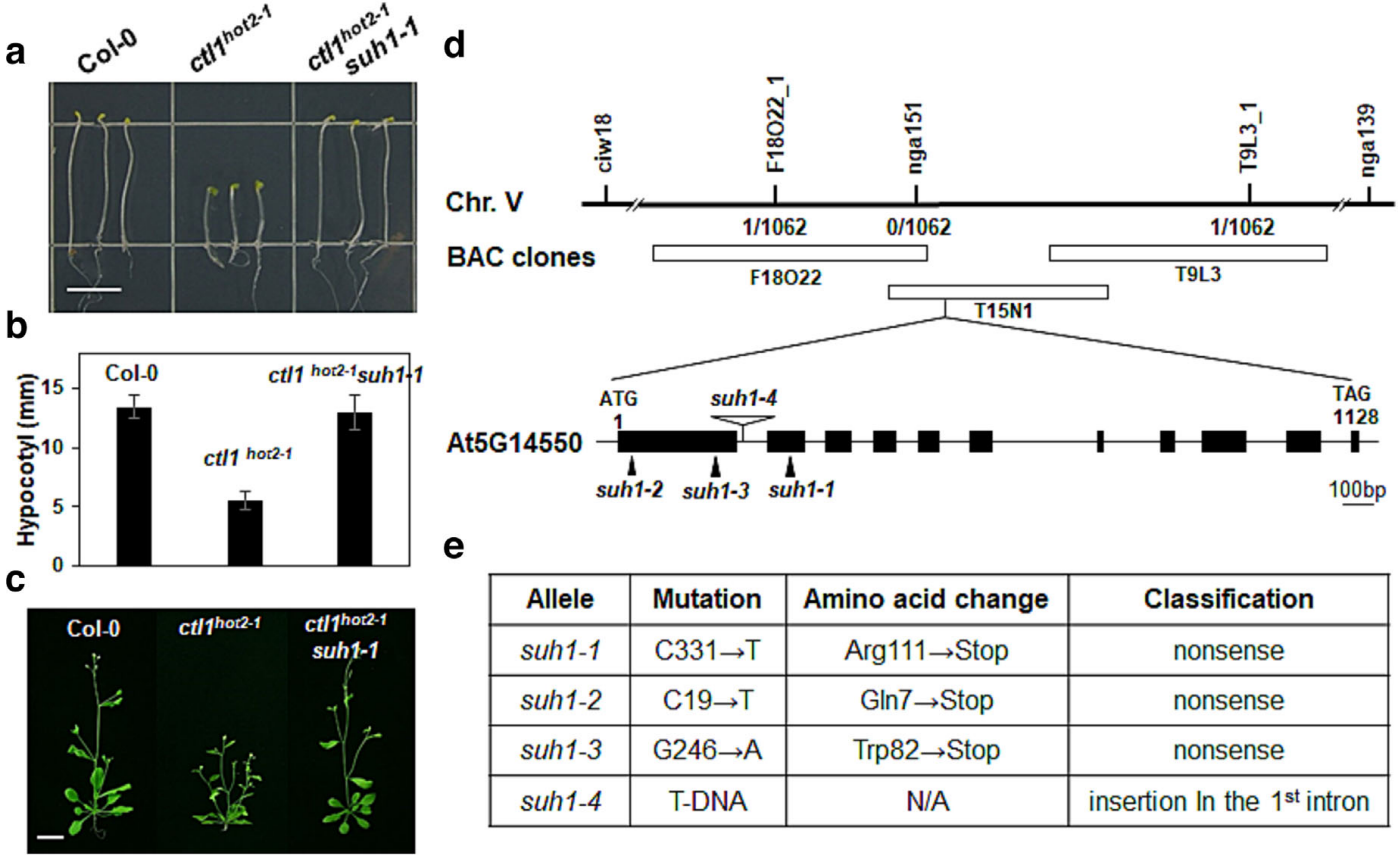
*SUH1* mutations restore growth defects in *ctl1* ^*hot2‐1*^ mutants. (a) Representative 5‐day‐old dark‐grown seedlings of Col‐0, *ctl1* ^*hot2‐1*^, and *ctl1* ^*hot2‐1*^ *suh1‐1* plants. Scale bar, 5 mm. (b) Quantification of hypocotyl length in etiolated seedlings shown in panel a. Data are expressed as the mean ± standard error (*SE)* of three replicates. (c) Representative 6‐week‐old light‐grown Col‐0, *ctl1* ^*hot2‐1*^, and *ctl1* ^*hot2‐1*^ *suh1‐1* plants. Scale bar, 1 cm. (d) Map‐based cloning of *SUH1*. *SUH1* was mapped to a region on chromosome 5 between two simple sequence length polymorphism (SSLP) markers, nga151 and T15N1_1, which is covered by three overlapping BAC clones: F18O22, T15N1, and T9L3. The recombinant numbers obtained from 536 F_2_ individual plants (1062 chromosomes) are indicated under each marker. The genomic structure of *SUH1* is shown with black boxes and lines indicating exons and introns. Solid arrowheads represent point mutations; T‐DNA insertion by an open arrowhead. (e) Descriptions and predicted molecular effects of the three *suh1* point mutations and the T‐DNA insertion. N/A indicates not applicable.

To investigate the molecular mechanisms underlying *CTL1*‐mediated cell wall assembly in *Arabidopsis*, a genetic screen was conducted on over 120,000 M_2_ ethyl methane sulfonate (EMS)‐mutagenized *ctl1* ^*hot2‐1*^ seedlings. *CTL1* mutations have been reported to inhibit *Arabidopsis* growth in both dark and light conditions. In particular, 5‐day‐old dark‐grown seedlings of *ctl1* ^*hot2‐1*^ exhibited a hypocotyl that is shorter than half that of the wild‐type seedlings. Therefore, identifying suppressor mutations that restore hypocotyl elongation in dark conditions enables mass screening of EMS‐mutagenized M_2_ seeds of *ctl1* ^*hot2‐1*^ in a Petri dish. Therefore, M_2_ seedlings were grown in dark conditions for 5 days, and seedlings whose hypocotyl elongated to wild‐type levels were selected. Finally, we isolated three independent suppressors that exhibited hypocotyl to wild‐type length in successive generations (Figure 1a,b). In addition, these suppressors also restored the abnormal morphology of light‐grown *ctl1* ^*hot2‐1*^ mutants, including the presence of numerous lateral branches and shorter stature of the aerial parts, to that of wild‐type plants (Figure 1c). Pair‐wise crosses of three suppressors demonstrated that the three mutations affected a single gene, leading to their designation as *suh1‐1*, *suh1‐2*, and *suh1‐3* (Table S1). Their crosses with *ctl1* ^*hot2‐1*^ mutants also revealed that the restoration of hypocotyl elongation is inherited in a recessive manner.

### 3.2 Map‐based cloning of *SUH1*

The *SUH1* locus was mapped to a 53‐kb segment on chromosome 5 (Figure 1d). Through sequencing analysis of *suh1‐1* mutants, we identified a premature stop codon caused by a substitution of Arg at position 111 in the second exon of *At5g14550*, which consists of 11 exons and 10 introns (Figure 1e). Nonsense mutations were also detected in *suh1‐2* and *suh1‐*3, where Gln (Cheng & Radhakrishnan, 2011) and Trp^82^ in the first exon of *At5g14550* were replaced with stop codons, respectively (Figure 1d,e). Moreover, we identified a fourth mutant allele, *suh1‐4*, which carried T‐DNA in the first intron of *At5g14550* (Figure 1d,e). No *SUH1* transcripts were detected in *suh1‐4* mutant plants (Figure S1A), and *suh1‐4* was confirmed to restore the growth defects of *ctl1* ^*hot2‐1*^ mutants under both dark and light conditions. The introduction of *SUH1* cDNA driven by its promoter in *ctl1* ^*hot2‐1*^ *suh1‐1* mutants resulted in a reversion to the *ctl1* ^*hot2‐1*^ phenotype under both dark and light conditions (Figure S1B,C), providing further evidence that supports the mutation in *SUH1* (*At5g14550*) is responsible for suppressing the *ctl1* ^*hot2‐1*^ phenotype.

Hématy et al. (2007) showed that mutations in *THE1*, which encodes a receptor‐like kinase, partially restore the short hypocotyl of etiolated *cesa6* ^*prc1‐1*^ carrying a null mutation in *CESA6* (resulting in reduced cellulose levels) and *pom1‐2* seedlings, which is another *CTL1* allele in *Arabidopsis*. To examine whether *suh1* can also restore the short hypocotyl of etiolated *cesa6* ^*prc1‐1*^ mutant, *suh1‐4 cesa6* ^*prc1‐1*^ double mutants were generated by crossing *suh1‐4* and *cesa6* ^*prc1‐1*^ mutants. When grown in the dark, the appearance of *suh1‐4 cesa6* ^*prc1‐1*^ mutant seedlings was indistinguishable from the *cesa6* ^*prc1‐1*^ mutants (Figure S2), suggesting that *suh1‐4* is unable to rescue the growth defects exhibited by *cesa6* ^*prc1‐1*^ mutants. This implies that *SUH1* is involved in cell wall assembly associated with *CTL1* rather than directly responding to the lack of a functional CESA6 in *Arabidopsis*.

### 3.3 *SUH1* mutations restore multiple defects caused by *ctl1* ^*hot2‐1*^

**FIGURE 2:**
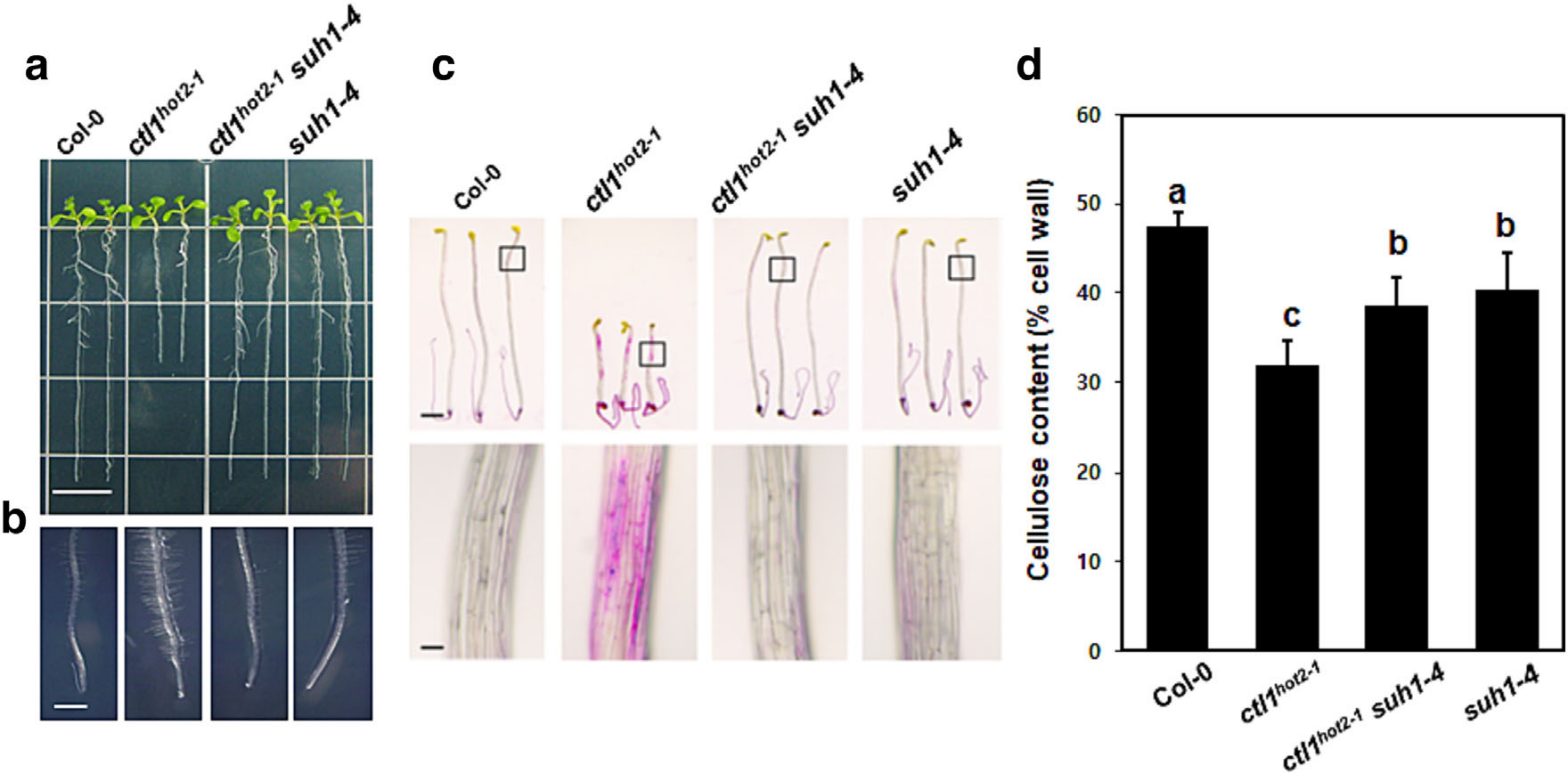
The *suh1* mutation restores the defects in *ctl1* ^*hot2‐1*^ mutants. Representative images of the primary root (a) and root hair (b) of 10‐day‐old wild‐type and mutant plants grown vertically on half‐strength MS medium. Scale bar, 1 cm (a) and 1 mm (b). (c) Five‐day‐old seedlings grown in the dark were examined for ectopic deposition of lignin using phloroglucinol–HCl staining. Strong red patches were detected only in *ctl1* ^*hot2‐1*^ seedlings. The area highlighted in the rectangular boxed region at the top panel was further enlarged in the bottom panel. Scale bar, 1 mm (top) and 50 μm (bottom). (d) Total cellulose contents (% cell wall) in inflorescence stems of 6‐week‐old plants. Data are expressed as the mean ± *SE* (11 samples). Statistically significant differences are indicated with different letters (one‐way ANOVA followed by Tukey's test, *P* < .05).

To examine the effect of *suh1* on the various defects linked to the *CTL1* mutation, we compared the phenotypes of plants carrying four combinations of *ctl1* ^*hot2‐1*^ and *suh1‐4*. First, we confirmed that *suh1‐4* restored the root growth of *ctl1* ^*hot2‐1*^ mutants to wild‐type levels (Figure 2a). As shown in Figure 2b, *suh1‐4* suppressed the increase in root hair density in *ctl1* ^*hot2‐1*^ plants; the root hair density of *ctl1* ^*hot2‐1*^ *suh1‐4* plants was indistinguishable from the wild‐type and *suh1‐4* plants. We also compared the growth of wild‐type and mutant plants in a growth chamber under long‐day conditions for 6 weeks (Figure S3A). The *suh1‐4* mutation clearly restored the semi‐dwarf and multiple branched defects exhibited by the *ctl1* ^*hot2‐1*^ mutants. However, the height of the *ctl1* ^*hot2‐1*^ *suh1‐4* and *suh1‐4* plants was slightly shorter, although not statistically significant, compared with the wild‐type plants (Figure S3B).

Next, we examined whether *suh1* could restore changes in cell wall composition and the abnormal cell shape demonstrated by *ctl1* ^*hot2‐1*^ mutants. First, to determine lignin content, 5‐day‐old seedlings of wild‐type and mutant plants grown in the dark were treated with phloroglucinol‐HCl, which stains lignin red (Figure 2c). Unlike *ctl1* ^*hot2‐1*^, which displayed dark‐red patches, no red patches were detected in the *ctl1* ^*hot2‐1*^ *suh1‐4* and *suh1‐4* mutant seedlings, similar to the wild‐type seedlings. Using the Updegraff assay (Kumar & Turner, 2015), we also found that the cellulose content in the *ctl1* ^*hot2‐1*^ mutants decreased to 66.9% of that in the wild‐type plants and that *suh1‐4* restored the cellulose content in the *ctl1* ^*hot2‐1*^ mutants to 81.6% of that in the wild‐type plants (Figure 2d). In addition to these restorations, further histochemical staining analyses revealed that the deposition patterns of pectin (Figure S4A) and arabinogalactan proteins (AGPs; Figure S4B) in the inflorescence stems of the *ctl1* ^*hot2‐1*^ *suh1‐4* mutants were indistinguishable from those in the *suh1‐4* and wild‐type plants, unlike the *ctl1* ^*hot2‐1*^ plants, which displayed ectopic depositions. These histochemical staining analyses clearly show that the *suh1‐4* mutation can recover their phenotype, although it is difficult to exclude the *suh1‐4* effect on lignin, pectin, and AGP synthesis. Cross‐sections of the mature stems showed that *suh1‐4* rescued the larger and irregularly shaped cells in the pith of the *ctl1* ^*hot2‐1*^ mutants to the wild‐type phenotype (Figure S4C). Moreover, we confirmed that *suh1‐4* also restored the reduced tolerance to high temperature (Figure S5A,B) and salinity stress (Figure S5C,D) of *ctl1* ^*hot2‐1*^ plants. Interestingly, *ctl1* ^*hot2‐1*^ *suh1‐4* and *suh1‐4* exhibited partial recovery when placed in half‐strength MS media containing 50‐mM NaCl compared with wild‐type plants. Contrastingly, the tolerance to high temperature was restored almost to wild‐type levels. These findings indicate similarities between defects in *suh1* and *ctl1* ^*hot2‐1*^ mutant plants, albeit to different degrees. Importantly, our results clearly demonstrate that the *suh1* mutation restores almost all defects caused by the *CTL1* mutation, at least partially or virtually, to wild‐type levels.

### 3.4 *suh1* alters the response to cellulose biosynthesis inhibitors

**FIGURE 3:**
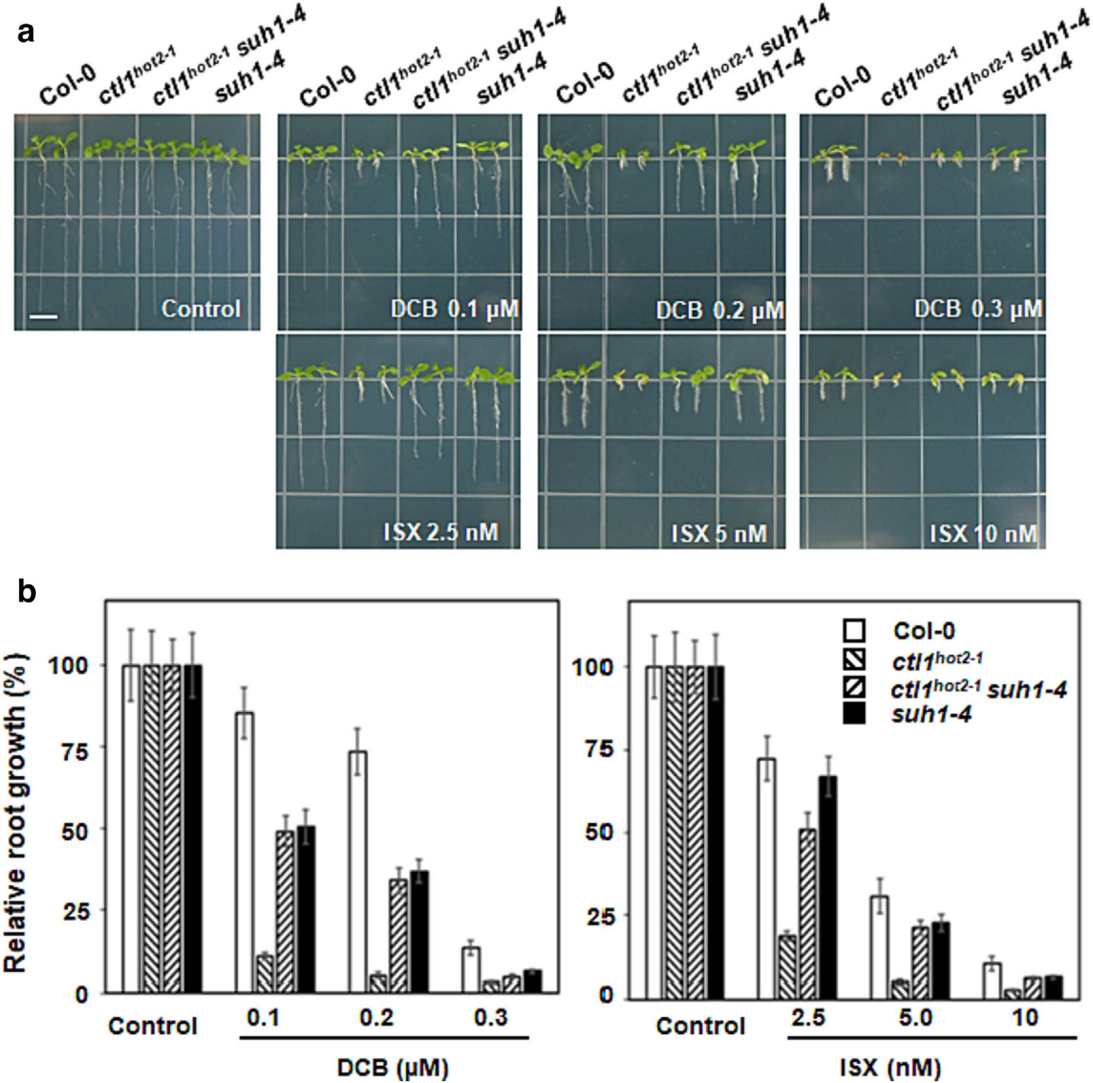
Responses to CBI treatment by wild‐type and mutant seedlings. Upon CBI treatment, the upper limit of root growth in *ctl1* ^*hot2‐1*^ *suh1‐4* plants is determined by the *suh1‐4* mutation. (a) Representative 10‐day‐old seedlings of wild‐type and mutant plants grown on half‐strength media containing different concentrations of DCB or ISX. Scale bar, .5 cm. (b) Quantification of root lengths of the seedlings described in (a). Data are presented as the mean ± *SE* of three replicates of 15 seedlings each.

The discovery that *suh1‐4* affects cellulose synthesis prompted us to investigate the response of wild‐type and mutant plants to chemicals that inhibit cellulose synthesis. Thus, we examined root growth in seedlings grown for 10 days in the light on half‐strength MS media supplemented with cellulose biosynthesis inhibitors (CBIs), such as 2,6‐dichlorobenzonitrile (DCB) and isoxaben (ISX). The primary root growth of wild‐type plants on the medium with the lowest concentration of CBIs was more than 70% of the control plants grown on the medium without CBIs. In contrast, the primary roots of the *ctl1* ^*hot2‐1*^ mutants barely grew under the same conditions (Figure 3). As expected, the primary roots of the *ctl1* ^*hot2‐1*^ *suh1‐4* mutants exhibited significant elongation at all inhibitor concentrations tested compared with the *ctl1* ^*hot2‐1*^ mutants, suggesting that *suh1‐4* partially restores root growth retardation in *ctl1* ^*hot2‐1*^ mutants grown on half‐strength MS media containing CBIs. Furthermore, *suh1‐4* exhibited root growth comparable to *ctl1* ^*hot2‐1*^ *suh1‐4*, which is shorter than the wild‐type plants at all concentrations of the CBIs. In addition, *suh1‐4* was more sensitive to DCB than to ISX, which induces CESA internalization. However, DCB affects microtubule‐associated proteins (MAPs) that play a key role in vesicle transport (Wormit et al., 2012). Therefore, these results suggest that *suh1* may be more sensitive to vesicle transport disruption rather than a lack of cellulose synthase due to CESA internalization.

### 3.5 *SUH1* encodes a Golgi‐localized type II membrane protein containing the Domain of Unknown Function 266

**FIGURE 4:**
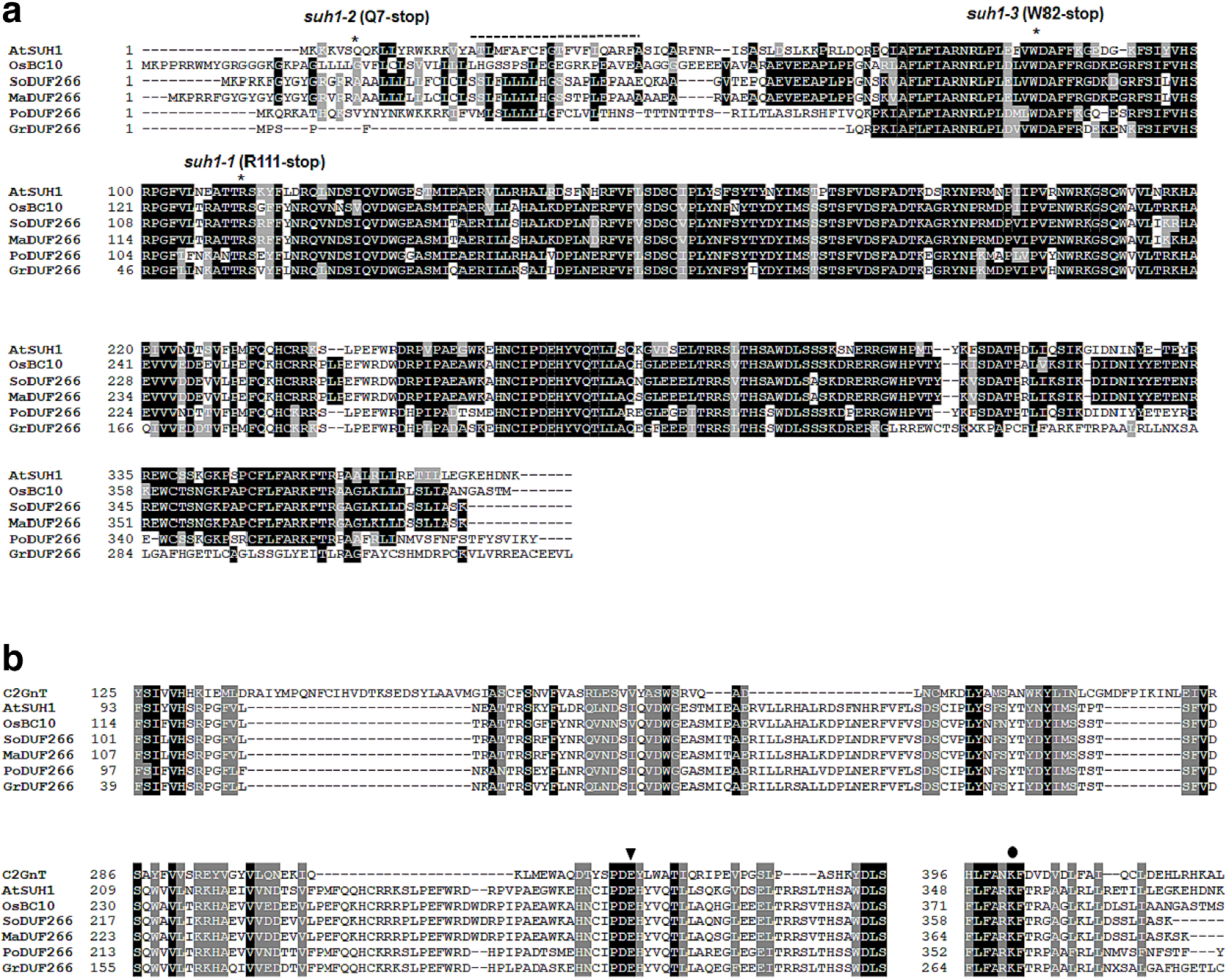
*SUH1* encodes a transmembrane protein containing a Domain of Unknown Function 266 (DUF266) that is highly similar to the GT14 protein. (a) Multiple alignment analysis of SUH1 and predicted DUF266‐containing proteins from plants. The predicted amino acid sequences from *Arabidopsis* (SUH1, B6IDH4), rice (BC10, Q65XS5), sorghum (So, C5Z134), maize (Ma, B4FL81), poplar (Pp, B9GHR6), and grape (Gr, A5AN07) were aligned using the ClustalW program (mobyle.pasteur.fr/cgi‐bin/portal.py). White letters on a black background indicate invariant residues; other conserved amino acids are shaded in gray. The dotted line indicates the predicted transmembrane region (amino acids 20–39), and the positions of *suh1* point mutations are indicated by asterisks. (b) Partial amino acid sequence alignment of DUF266 protein members and the human Core2 1,6 β‐*N*‐acetylglucosaminyltransferase (Q02742) in the GT14 family. Bioinformatic studies revealed structural similarities and invariant amino acid residues conserved between DUF266 proteins and leukocyte type core‐2 β‐1,6‐*N*‐acetylglucosaminyltransferase (C2GnT‐L), a member of the GT14 family, suggesting a distant relationship between DUF266 proteins and GT14. The first region presumably corresponds to the Rossmann‐fold motif for nucleotide binding. The second region is a structural domain that interacts with the donor and acceptor substrates. The conservation of the putative catalytic residue Glu‐320 in C2GnT in DUF266 members is particularly interesting. The putative catalytic residue (Glu‐320 in C2GnT) is marked with an inverted triangle. The third region at the C‐terminus of the catalytic domain shows three invariant residues. One of them, Lys‐401 in C2GnT (marked with a circle), which is expected to interact with the diphosphate group of the nucleotide sugar, is also conserved in the DUF266 family.

The *SUH1* cDNA has an open reading frame of 1134 nucleotides, which encodes a polypeptide of 377 amino acids with a predicted molecular mass of 44.6 kDa. Sequence analyses using Pfam (Finn et al., 2006) revealed an N‐terminal transmembrane domain (TM, amino acids 20–39) followed by a C‐terminus that contains the Domain of Unknown Function 266 (DUF266, amino acids 66–322). The multiple alignment of various DUF266‐containing proteins showed that SUH1 is most similar to the rice BC10 (Figure 4a). Bioinformatic analyses suggest that DUF266‐containing proteins have structural similarities to the leukocyte core‐2 β1, 6N‐acetylglucosaminyltransferase (C2GnT‐L; Figure 4b), which is a member of the glycosyltransferase 14 (GT14) family (Hansen et al., 2009). DUF266‐containing proteins are structurally related to the GT14 family and have been found in almost all plants (Ye et al., 2011). However, the study of *Brittle Culm 10* (*OsBC10*), which encodes a type II membrane protein containing DUF266 in rice, was the first report on their functional characterization (Zhou et al., 2009). The catalytic region of human C2GnT contains three functional domains (Hansen et al., 2009; Figure 4b). The first domain is the Rossmann‐type nucleotide‐binding domain (amino acids 125–225 in C2GnT), followed by the substrate‐interaction domain (amino acids 286–345 in C2GnT), and the final domain binds to the diphosphate group in the nucleotide (amino acids 396–424 in C2GnT). We identified counterparts exhibiting high similarity to the three functional domains in C2GnT in the DUF266 proteins (Figure 4b). It is notable that the Glu^320^ and Lys^401^ residues in C2GnT, which are involved in catalysis and nucleotide binding, are also identified in DUF266 proteins. These residues are indicated by an asterisk and a circle, respectively (Figure 4b).

### 3.6 *SUH1* is the *Arabidopsis* ortholog of the rice *BC10* gene

**FIGURE 5:**
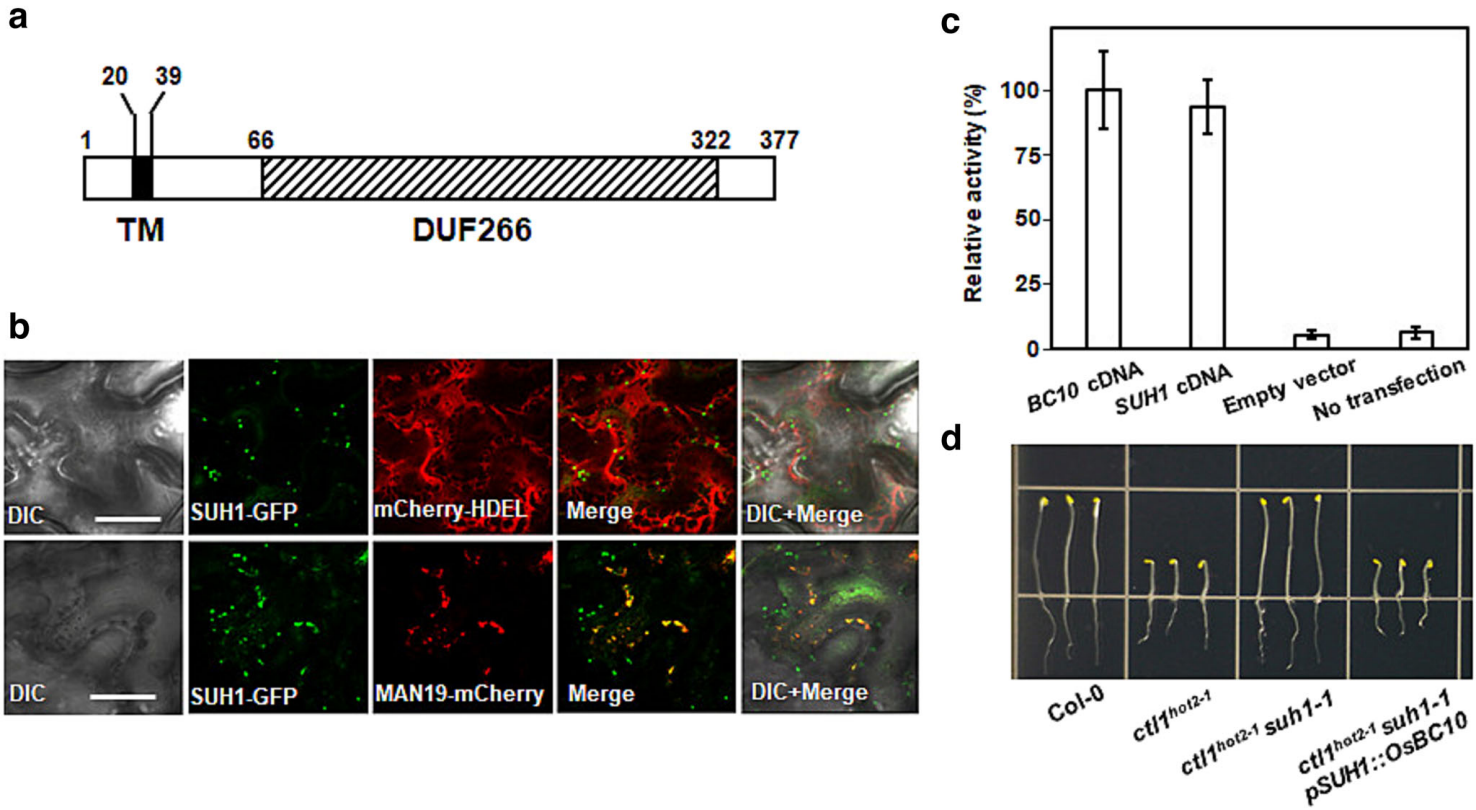
*SUH1* is the *Arabidopsis* ortholog of the rice *BC10* gene encoding a Golgi‐localized type II glycosyltransferase. (a) The deduced protein domain structure of SUH1. TM (black box) and DUF266 (hatched box) indicate the transmembrane domain and Domain of Unknown Function 266, respectively. The numbers denote the amino acid positions. (b) Confocal images of tobacco leaf epidermal cells transiently co‐expressing SUH1‐GFP with the ER marker mCherry–HDEL or the Golgi marker MAN49‐mCherry. SUH1‐GFP and MAN49‐mCherry co‐expression labeling of Golgi vesicles shows co‐localization. GFP and mCherry produce green and red fluorescence, respectively. The merged images of GFP and mCherry presented yellow fluorescence with and without a DIC (differential interference contrast) background. Scale bar, 50 μm. (c) C2GnT assay in CHO cells. C2GnT activity was determined using PNP‐oligosaccharide as an acceptor. The C2GnT activities of each construct and control are expressed as a percentage of BC10. Data are expressed as the mean ± *SE* from three independent measurements. (d) Complementation of the short‐hypocotyl phenotype of dark‐grown *ctl1* ^*hot2‐1*^ *suh1‐1* mutants with rice *BC10* driven by the *SUH1* promoter.

OsBC10, which has a protein structure most similar to SUH1 (Figure 5a), is a Golgi‐localized type II membrane protein with about 1% of human C2GnT activity in Chinese hamster ovary (CHO) cells (Zhou et al., 2009). This finding led us to perform the following functional characterization. Before conducting functional analyses, we confirmed that the introduction of the *SUH1‐GFP* construct under the *SUH1* promoter restored the *ctl1* ^*hot2‐1*^ *suh1‐4* phenotype to that of the *ctl1* ^*hot2‐1*^ mutants (Figure S6). This result suggests that SUH1‐GFP retains the SUH1 biological functions. To determine the subcellular localization of SUH1, the SUH1‐GFP fusion protein was transiently co‐expressed with MAN49‐mCherry or mCherry‐HDEL in tobacco leaf epidermal cells. SUH1‐GFP displayed a punctate localization pattern (Figure 5b), which exactly overlapped with the known Golgi marker, MAN49‐mCherry, but not with the mCherry‐HDEL localized in the endoplasmic reticulum lumen (Nelson et al., 2007). These findings suggest that *SUH1*, similar to the rice *OsBC10*, encodes a Golgi‐localized protein containing a DUF266 domain.

Next, we performed the C2GnT assay using CHO cells as described previously (Bierhuizen & Fukuda, 1992). The plasmid carrying either the *BC10‐GFP* or *SUH1‐GFP* construct was transfected into CHO cells. As shown in Figure 5c, CHO cells expressing *SUH1‐GFP* displayed C2GnT activity at levels comparable to the cells transfected with *BC10‐GFP*. However, the C2GnT activity was barely detectable in nontransfected CHO cells, or CHO cells transfected with an empty vector. To further assess the potential of *SUH1* as an ortholog of *OsBC10*, *BC10* cDNA under the control of the *SUH1* promoter (*pSUH1::BC10*) was introduced into *ctl1* ^*hot2‐1*^ *suh1‐4* mutant plants (Figure S1B). As anticipated, the growth phenotype of *ctl1* ^*hot2‐1*^ *suh1‐4* plants expressing *OsBC10* was indistinguishable from the *ctl1* ^*hot2‐1*^ mutant plants under both dark (Figure 5d) and light conditions (Figure S1C). These findings demonstrate that *SUH1*, an *Arabidopsis* ortholog of *OsBC10*, encodes a Golgi‐localized type II membrane protein with glycosyltransferase activity.

### 3.7 *SUH1* is predominantly expressed in tissues associated with secondary cell wall deposition

**FIGURE 6:**
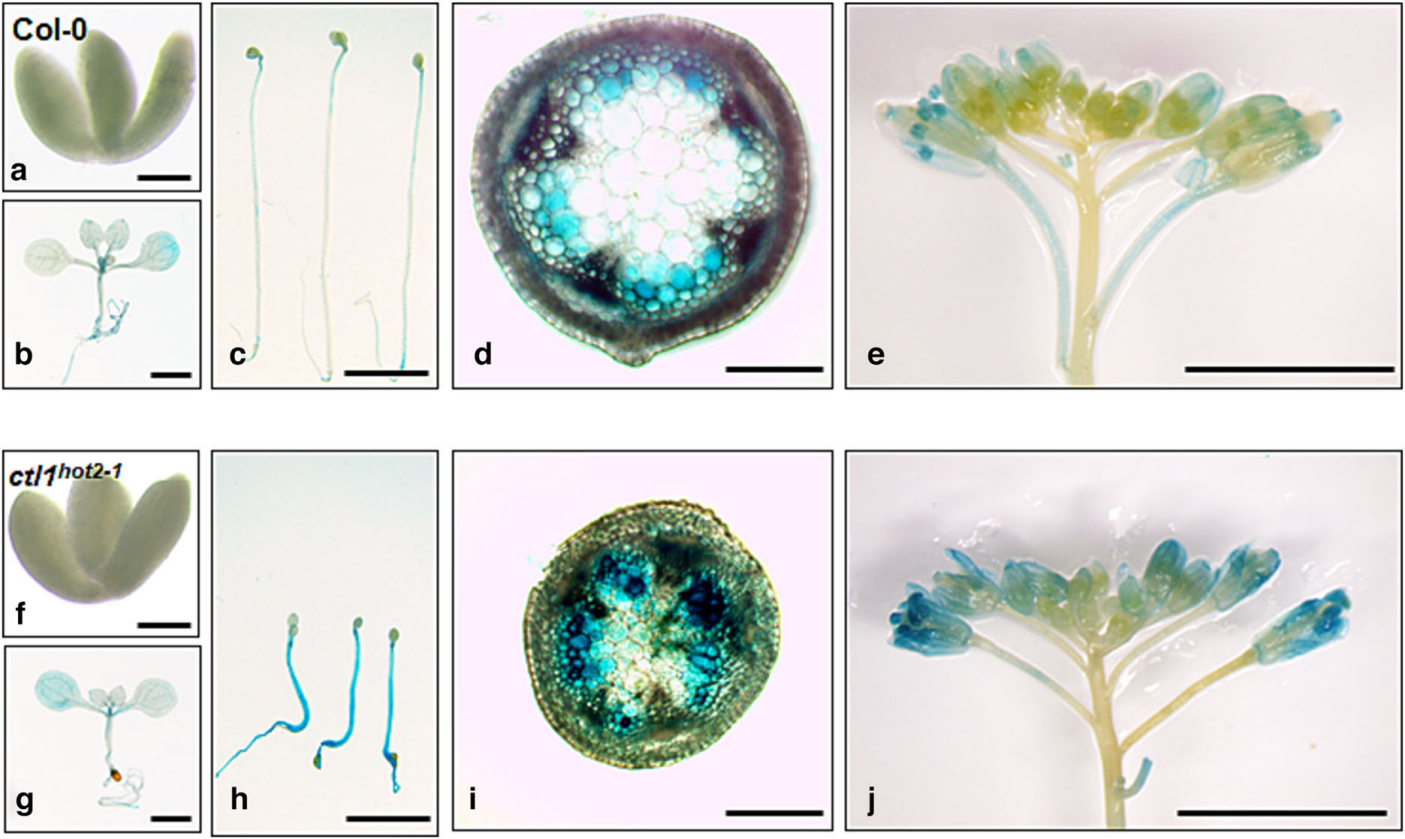
Histochemical assay for GUS activity in transgenic plants with the *pSUH1::GUS* construct. Representative images of Col‐0 (a–e) and *ctl1* ^*hot2‐1*^ (f–g) plants from 15 lines with three replicates. (a,f) The embryos are from mature seeds. (b,g) Ten‐day‐old seedlings grown in the light. (c,h) Five‐day‐old seedlings grown in the dark. (d, i) Hand‐cut sections of inflorescence stem of 6‐week‐old plants. (e,j) Flowers of 6‐week‐old plants. Scale bars, 200 μm (a, b, c, f, g, and h) and 5 mm (d, e, i, and j).

To investigate the *SUH1* expression pattern, we generated transgenic wild‐type and *ctl1* ^*hot2‐1*^ mutant plants expressing the β‐glucuronidase (GUS) reporter gene driven by the 1060 bp *SUH1* promoter region located upstream of the ATG start codon (*pSUH1::GUS*). In the wild‐type plants carrying the *pSUH1::GUS* construct, GUS activity was barely detectable in embryos, 5‐day‐old dark‐grown seedlings, and 10‐day‐old light‐grown seedlings; indeed, deposition of secondary cell walls was minimized during these early developmental stages (Figure 6a–c). However, a strong GUS activity was detected in the interfascicular fibers, xylem cells in the inflorescence stems (Figure 6d), and mature anthers (Figure 6e), where the secondary cell walls have been reported to be deposited (Taylor‐Teeples et al., 2015). Similar to the wild‐type plants, *ctl1* ^*hot2‐1*^ showed little *pSUH1*‐driven GUS activity in embryos (Figure 6f) and 10‐day‐old seedlings grown in the light (Figure 6g), where lignin is not deposited. However, in contrast to the wild‐type plants, *ctl1* ^*hot2‐1*^ exhibited strong GUS staining in etiolated seedlings (Figure 6h) and pith cells (Figure 6i), where ectopic lignin is deposited due to the *ctl1* ^*hot2‐1*^ mutation. An increase in GUS staining due to *ctl1* ^*hot2‐1*^ was also found in the flowers (Figure 6e,j). These results suggest that the *SUH1* expression is associated with tissues that deposit the secondary cell wall and is enhanced by alterations in cell wall composition caused by the *ctl1* ^*hot2‐1*^ mutation.

## 4 DISCUSSION

Mutations in *CTL1* cause several defects in *Arabidopsis*, including growth retardation and changes in cell wall composition, due to reduced cellulose contents (Hauser et al., 1995; Hong et al., 2003; Zhong et al., 2002). Our findings demonstrate the *suh1*‐mediated recovery of multiple defects caused by *ctl1* ^*hot2‐1*^ mutation, including growth and development, cell wall composition, and abiotic stresses. Notably, we showed that *suh1‐4* affects the cellulose content (Figure 2d), response to salinity stress (Figure S5B), and root growth under CBI treatments (Figure 3), although to a lesser extent. These results suggest that *suh1‐4* also displays similarities to defects in *ctl1* ^*hot2‐1*^ involving cell wall assembly in *Arabidopsis*. It has previously been posited that if a suppressor mutation displays a phenotype similar to the original mutation, the two gene products likely regulate the same step in a multistep pathway (Prelich, 1999). Therefore, considering the genetic relationship between *CTL1* and *SUH1*, it is probable that these two genes are involved in the same process in a multistep pathway. Moreover, we revealed that *SUH1* encodes a Golgi‐localized type II membrane protein with glycosyltransferase activity. These observations provide further insights into the cell wall synthesis mechanism mediated by *CTL1* in *Arabidopsis*.

SUH1 was delivered to the Golgi complex similar to BC10 of rice (Figure 5b). At the same time, CTL1 was found in various organelles on the secretory pathway from the Golgi complex to the cell wall in *Arabidopsis* (Sánchez‐Rodríguez et al., 2012). Therefore, the assumption that CTL1 and SUH1 regulate the same step in a multistep pathway suggests that CTL1‐mediated processing possibly occurs in the Golgi complex, where the two proteins coexist, rather than in the apoplastic region where CTL1 is delivered alone. However, our results cannot exclude the possible role of apoplastic CTL1 suggested in a previous report (Sánchez‐Rodríguez et al., 2012). Interestingly, mutations in *BC15*/*OsCTL1* in rice, the closest ortholog of *AtCTL1*, affect cell wall synthesis by reducing cellulose synthesis, while its product is also targeted to the Golgi complex (Wu et al., 2012). These results also make it extremely interesting to investigate whether the same genetic relationship between *ctl1* ^*hot2‐1*^ and *suh1* in *Arabidopsis* would also be observed between *bc10* and *bc15* in rice.

Elucidating the substrates of CTL1 is important for understanding its molecular mechanism. Assuming that CTL1 and SUH1 regulate the same step in a multistep pathway, we consider understanding the possible substrates of SUH1 to be equally important. We showed that SUH1 contains DUF266, which is known to be related to the GT14 family (Figure 4b), and exhibits C2GnT activity (Figure 5c). In animal cells, C2GnT is implicated in the elongation of the core 2 *O*‐glycan branch of mucin, analogous to AGP glycans in plants (Basu et al., 2013; Cheng & Radhakrishnan, 2011). The side chain in the AGP glycans is also documented to be elongated by members of the GT14 family in *Arabidopsis* (Dilokpimol & Geshi, 2014; Knoch et al., 2013). Therefore, we suggest the possibility that SUH1 and CTL1 collaboratively regulate the glycosylation of AGPs in *Arabidopsis*, probably by promoting and blocking/degrading the same glycans of AGPs, respectively.

Finally, while *AtCTL1* expression is relatively strong in almost all tissues (Hossain et al., 2010), *SUH1* is predominantly expressed in interfascicular fibers and xylems with secondary cell walls (Figure 6a–e). In particular, *ctl1* ^*hot2‐1*^ causes increased expression of *SUH1* along with lignin accumulation in etiolated hypocotyl (Figure 6h) and stem pith cells (Figure 6i), which are not observed in wild‐type plants (Figure 6c,d). These findings suggest that *SUH1* transcripts are upregulated by an aberrant deposition of cell wall components caused by *ctl1* ^*hot2‐1*^. Therefore, to further understand the roles of *CTL1* in cell wall synthesis, it is essential to identify its endogenous substrates and elucidate how cell wall integrity affects the expression of *SUH1*. Overall, these results strongly reveal that *suh1* mutations suppress *ctl1* ^*hot2‐1*^‐induced defects in *Arabidopsis*. Additionally, the genetic relationship between these two mutations suggests that CTL1 and SUH1 may regulate the same step in a multistep pathway, indicating that they may share substrates. Therefore, further characterizations of *SUH1* are essential for a comprehensive understanding of cell wall synthesis in *Arabidopsis*.

## AUTHOR CONTRIBUTIONS

*Design and project management*: Suk‐Whan Hong. *Experiments and data analyses*: Nguyen Thi Thuy, Hyun‐Jung Kim, and Suk‐Whan Hong. *Writing and editing*: Nguyen Thi Thuy, Hyun‐Jung Kim, and Suk‐Whan Hong.

## CONFLICT OF INTEREST STATEMENT

The Authors did not report any conflict of interest.

## CONFLICT OF INTEREST STATEMENT

The authors have no conflicts of interest to declare.

## Supporting information


**Data S1.** Peer review

**Table S1.** Genetic analysis of the effect of the *suh1* mutation on hypocotyl elongation in the dark. Seeds (*N*_*total*_) were planted on half‐strength MS medium. The numbers of *ctl1* ^*hot2–1*^ *suh1‐1* (*N*_wt_) and *ctl1* ^*hot2–1*^ (*N*_*hot2–1*_) mutants were determined after growing at 22 °C in the dark for 5 days. F_1_ and F_2_ refer to the progeny generated by crossing the *ctl1* ^*hot2–1*^ *suh1‐1* mutants with the *ctl1* ^*hot2–1*^ mutants and the progeny obtained through self‐fertilization of F_1_ plants, respectively. ^a^All crosses are shown as “female parent x male parent”. ^b^A hypocotyl length of 9.5–12.5 mm was defined as the wild‐type phenotype and 4.0–7.0 mm was defined as the *hot2–1* phenotype. ^c^The Chi‐square (χ^2^) values were calculated for the expected segregation ratio 3:1.
**Table S2.** List of primers used in this research.
**Figure S1.** Complementation of *ctl1* ^*hot2–1*^ *suh1‐4* mutant plants. (A) RT‐PCR amplification of *SUH1* transcripts in wild‐type and *suh1‐4* mutant plants. *Actin2* was used as a loading control. (B) Schematic representation of the *pSUH1::SUH1* and *pSUH1::OsBC10* constructs used for complementation tests. The full‐length cDNAs of *SUH1* and *BC10* were placed under the control of the *SUH1* promoter sequence (1,060 bp) in the binary vector pBI121. (C) Five‐day‐old dark‐grown seedlings (top) and six‐week‐old mature plants (bottom) of Col‐0, *ctl1* ^*hot2–1*^, *ctl1* ^*hot2–1*^ *suh1‐4*, and two transgenic *ctl1* ^*hot2–1*^ *suh1‐4* plants carrying either the *pSUH1::SUH1* or *pSUH1::OsBC10* construct. The scale bars at the top and bottom represent 5 mm and 15 mm, respectively.
**Figure S2.** Genetic interactions between *suh1‐4* and *cesa6* ^*prc1–1*^. The *suh1‐4* mutation does not restore the growth defect of *cesa6* ^*prc1–1*^. (A) Five‐day‐old dark‐grown seedlings. (B) Six‐week‐old light‐grown plants of the genotypes Col‐0, *suh1‐4*, *cesa6* ^*prc1–1*^, and *cesa6* ^*prc1–1*^ *suh1‐4*. Scale bars, 5 mm (A), 15 mm (B).
**Figure S3.** Growth phenotypes of wild‐type and mutant plants. (A) Wild‐type and mutant plants were grown in the soil inside a growth chamber (16 h light/8 h dark, 22 °C/18 °C cycle under a light density of 120 μmol m^−2^ s^−1^) for 6 weeks before being photographed. Scale bars, 15 mm. (B) Quantification of heights of wild‐type and mutant plants described in (A). Data are presented as the mean ± *SE* of three replicates of 15 seedlings each. Statistically significant differences are indicated with different letters (one‐way ANOVA followed by Tukey's test, P < .05).
**Figure S4.** Comparison of stem characteristics of wild‐type and mutant plants. (A, B) Hand‐cut sections of the inflorescence stem of 6‐week‐old plants were stained with toluidine blue (A), or β‐glucosyl Yariv (β‐GlcY) reagent (B). Toluidine blue stains the cell wall containing lignin and pectin in blue‐green and pink, respectively. β‐GlcY stains arabinogalactan proteins (AGPs) in reddish brown. (C) Transverse sections of the inflorescence stems of 6‐week‐old wild‐type and mutant plants were stained by toluidine blue. Pith (Pi), vascular bundle (VB), interfascicular fiber (IF), and cortex (Co) are visible. Sections were prepared at the first internode of the stem. Scale bar, 200 μm.
**Figure S5.** Responses of wild‐type and mutant plants to heat and salt stress. (A) Thermotolerance assay of wild‐type and mutant plants. Seedlings were grown in the dark at 2.5 days old and pre‐treated at 38 °C for 90 min, then at 22 °C for 120 min. Seedlings were further stressed at 45 °C for 120 min and grown at 22 *°C* for 2.5 days before photographing. Seedlings grown in the dark for 5 days at 22 °C were used as controls. Scale bars, 5 mm. (B) Quantitative assessment of acquired thermotolerance of the seedlings shown in panel (A). The hypocotyl elongation levels in the seedlings subjected to the thermotolerance assay are expressed as a percentage of those in the seedlings of the same genotype grown for 5 days at 22 °C in the dark (controls). White and black bars indicate hypocotyl elongation under control and heat shock conditions, respectively. An asterisk indicates no elongation after heat shock, which was observed in *ctl1* ^*hot2–1*^ mutants. The mean and *SE* were calculated using data from three independent measurements, each with at least 15 seedlings. Scale bar, 1 cm. (C) Salt tolerance of wild‐type and mutant plants as measured by root growth. Three‐day‐old seedlings grown vertically in half‐strength MS medium were transferred to a medium containing indicated concentrations of NaCl and grown for 7 days. (D) A quantitative assessment of the salt sensitivity of the seedlings shown in panel (C). The mean and *SE* were calculated using data from three independent measurements, each with at least 15 seedlings. Root growth under saline conditions was expressed as a percentage of those grown under non‐stress conditions.
**Figure S6.** Complementation of *ctl1* ^*hot2–1*^ *suh1‐4* plants by the introduction of the *SUH1‐GFP* construct. Five‐day‐old dark‐grown seedlings (A) and six‐week‐old light‐grown plants (B) of the genotypes Col‐0, *ctl1* ^*hot2–1*^, *ctl1* ^*hot2–1*^ *suh1‐4*, and two transgenic *ctl1* ^*hot2–1*^ *suh1‐4* plants carrying either empty vector and the *pSUH1::SUH1‐GFP* construct. Scale bars, 5 mm (A), 15 mm (B).

## Data Availability

All data are provided in the main text or Supporting Information.
