# Supplementary material for: Antagonistic functions of CTL1 and SUH1 mediate cell wall assembly in Arabidopsis
Source: Plant Direct. 2024 Mar 23;8(3):e580. doi: 10.1002/pld3.580 (PMC10960159; doi:10.1002/pld3.580)
Supplement: Supplementary file 2 — Table S1. Genetic analysis of the effect of the suh1 mutation on hypocotyl elongation in the dark. Seeds (N total ) were planted on half‐strength MS medium. The numbers of ctl1 hot2–1 suh1‐1 (N wt) and ctl1 hot2–1 (N hot2–1 ) mutants were determined after growing at 22 °C in the dark for 5 days. F1 and F2 refer to the progeny generated by crossing the ctl1 hot2–1 suh1‐1 mutants with the ctl1 hot2–1 mutants and the progeny obtained through self‐fertilization of F1 plants, respectively. aAll crosses are shown as “female parent x male parent”. bA hypocotyl length of 9.5–12.5 mm was defined as the wild‐type phenotype and 4.0–7.0 mm was defined as the hot2–1 phenotype. cThe Chi‐square (χ2) values were calculated for the expected segregation ratio 3:1. Table S2. List of primers used in this research. Figure S1. Complementation of ctl1 hot2–1 suh1‐4 mutant plants. (A) RT‐PCR amplification of SUH1 transcripts in wild‐type and suh1‐4 mutant plants. Actin2 was used as a loading control. (B) Schematic representation of the pSUH1::SUH1 and pSUH1::OsBC10 constructs used for complementation tests. The full‐length cDNAs of SUH1 and BC10 were placed under the control of the SUH1 promoter sequence (1,060 bp) in the binary vector pBI121. (C) Five‐day‐old dark‐grown seedlings (top) and six‐week‐old mature plants (bottom) of Col‐0, ctl1 hot2–1 , ctl1 hot2–1 suh1‐4, and two transgenic ctl1 hot2–1 suh1‐4 plants carrying either the pSUH1::SUH1 or pSUH1::OsBC10 construct. The scale bars at the top and bottom represent 5 mm and 15 mm, respectively. Figure S2. Genetic interactions between suh1‐4 and cesa6 prc1–1 . The suh1‐4 mutation does not restore the growth defect of cesa6 prc1–1 . (A) Five‐day‐old dark‐grown seedlings. (B) Six‐week‐old light‐grown plants of the genotypes Col‐0, suh1‐4, cesa6 prc1–1 , and cesa6 prc1–1 suh1‐4. Scale bars, 5 mm (A), 15 mm (B). Figure S3. Growth phenotypes of wild‐type and mutant plants. (A) Wild‐type and mutant plants were grown in the soil inside a growth [file PLD3-8-e580-s002.docx]

**Supporting Information**

**
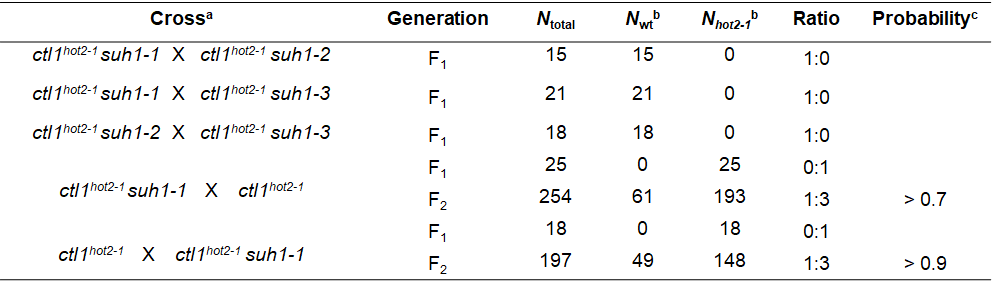
**

**Table S1. Genetic analysis of the effect of the *suh1* mutation on hypocotyl elongation in the dark.** Seeds (*N_total_*) were planted on half-strength MS medium. The numbers of *ctl1^hot2-1^ suh1-1* (*N*_wt_) and *ctl1^hot2-1^* (*N_hot2-1_*) mutants were determined after growing at 22 °C in the dark for 5 days. F_1_ and F_2_ refer to the progeny generated by crossing the *ctl1^hot2-1^ suh1-1* mutants with the *ctl1^hot2-1^* mutants and the progeny obtained through self-fertilization of F_1_ plants, respectively. ^a^All crosses are shown as “female parent x male parent”. ^b^A hypocotyl length of 9.5–12.5 mm was defined as the wild-type phenotype and 4.0–7.0 mm was defined as the *hot2-1* phenotype. ^c^The Chi-square (χ^2^) values were calculated for the expected segregation ratio 3:1.

**Table S2. List of primers used in this research.**

| **Primer's name** | **Primer sequences (5’ to 3’)** | **Note** |
| --- | --- | --- |
| **Molecular marker** |  | **Location** |
| nga248(F) | TCTGTATCTCGGTGAATTCTCC | chromosome 1 |
| nga248(R) | TACCGAACCAAAACACAAAGG |  |
| nga6(F) | ACCCAAGTGATCGCCACC | chromosome 1 |
| nga6(R) | AACCAAGGCACAGAAGCG |  |
| nga1126(F) | CGCTACGCTTTTCGGTAAAG | chromosome 2 |
| nga1126(R) | GCACAGTCCAAGTCACAACC |  |
| nga168(F) | tcgtctactgcactgccg | chromosome 2 |
| nga168(R) | gaggacatgtataggagcctcg |  |
| nga162(F) | CATGCAATTTGCATCTGAGG | chromosome 3 |
| nga162(R) | CTCTGTCACTCTTTTCCTCTGG |  |
| nga172(F) | CATCCGAATGCCATTGTTC | chromosome 3 |
| nga172(R) | AGCTGCTTCCTTATAGCGTCC |  |
| nga8(F) | TGGCTTTCGTTTATAAACATCC | chromosome 4 |
| nga8(R) | GAGGGCAAATCTTTATTTCGG |  |
| nga1107(F) | CGACGAATCGACAGAATTAGG | chromosome 4 |
| nga1107(R) | GCGAAAAAACAAAAAAATCCA |  |
| ciw10 (F) | CCACATTTTCCTTCTTTCATA | chromosome 5 |
| ciw10 (R) | CAACATTTAGCAAATCAACTT |  |
| ciw18 (F) | AACACAACATGGTTTCAGT | chromosome 5 |
| ciw18 (R) | GCCGTTTGTCTCTTCAC |  |
| nga225(F) | TCTCCCCACTAGTTTTGTGTCC | chromosome 5 |
| nga225(R) | GAAATCCAAATCCCAGAGAGG |  |
| nga106(F) | TGCCCCATTTTGTTCTTCTC | chromosome 5 |
| nga106(R) | GTTATGGAGTTTCTAGGGCACG |  |
| nga225(F) | TCTCCCCACTAGTTTTGTGTCC | chromosome 5 |
| nga225(R) | GAAATCCAAATCCCAGAGAGG |  |
| nga249(F) | GGATCCCTAACTGTAAAATCCC | chromosome 5 |
| nga249(R) | TACCGTCAATTTCATCGCC |  |
| nga76(F) | AGGCATGGGAGACATTTACG | chromosome 5 |
| nga76(R) | GGAGAAAATGTCACTCTCCACC |  |
| nga139(F) | GGTTTCGTTTCACTATCCAGG | chromosome 5 |
| nga139(R) | AGAGCTACCAGATCCGATGG |  |
| nga106(F) | gttatggagtttctagggcacg | chromosome 5 |
| nga106(R) | tgccccattttgttcttctc |  |
| nga151(F) | CAGTCTAAAAGCGAGAGTATGATG | chromosome 5 |
| nga151(R) | GTTTTGGGAAGTTTTGCTGG |  |
| Ca72(F) | CCCAGTCTAACCACGACCAC | chromosome 5 |
| Ca72(R) | AATCCCAGTAACCAAACACACA |  |
| T15N1_1(F) | cagtctaaaagcgagagtatg | chromosome 5  (BAC:T15N1) |
| T15N1_1(R) | gttttgggaagttttgctgg |  |
| MEX10_1(F) | cgtcagggtgctgcttttctc | chromosome 5 (BAC:MXE10) |
| MEX10_1(R) | gtgcctgcacattgatcaccatc |  |
| MAC12_1(F) | ATGTCGACTattgacttgag | chromosome 5 (BAC:MAC12) |
| MAC12_1(R) | ttcagcttcaatgattaaac |  |
| MUA22_1(F) | ggagagactgatggacgccatttg | chromosome 5 (BAC:MUA22) |
| MUA22_1(R) | gtcctcatcaaggggctgcagagg |  |
| MSH12_1(F) | ggactgttgctttattattc | chromosome 5 (BAC:MSH12) |
| MSH12_1(R) | ttcaaagctggataacaga |  |
| T9L3_1 (F) | GTAacgtatgcatggtttg | chromosome 5 (BAC: T9L3) |
| T9L3_1 (R) | aagttttggttagattacac |  |
| F18O22_1(F) | gcaaagccgttgatatgttag | chromosome 5 (BAC:F18O22) |
| F18O22_1 (R) | gtcctctgactagaaagaggc |  |
| F18O22_2(F) | cgtcgacgttaatttcgtcgg | chromosome 5 (BAC:F18O22) |
| F18O22_2(R) | gtaataatcagcgttcgttcg |  |
| **Constructs** | **(Note: the underlined nucleotides indicate the restriction site for cloning)** | **Construct name** |
| SUH1-SmaI | GCCCGGGcgattttctgtgtggatccaa | *SUH1* cDNA |
| SUH1-SacI | GGAGCTCctcttggttatcttacgtacc |  |
| OsBC10-Smal | GCCCGGGACGGTGGTCGCCGGAGATGAA | *OsBC10* cDNA |
| OsBC10-SacI | GGAGCTCTGCAAGCTGGCAGTATTCTGC |  |
| pSUH1-Xbal | GTCTAGAtttggatccacacagaaaatc | *pBI121-pSUH1::GUS* |
| pSUH1-HindIII | GAAGCTTTGACCTGAGAACACATGAAAC |  |
| SUH1-GFP-NheI | GCGCTAGCATGAAGAAGAAGGTGTCTCAG | *p1300-35S-SUH1::GFP* |
| SUH1-GFP-BamHI | GCGGATCCCTTATTGTCATGCTCTTTGCC |  |
| **Genotyping** |  | **Purpose** |
| suh1-4(F) | TCTCACTCTCCAAGTCTCCTC | Genotyping *suh1-4* |
| suh1-4(R) | CACGTTCTGCTTCAATCATGG |  |
| SAIL T-DNA primer | GCCTTTTCAGAAATGGATAAATAGCCTTGCTTCC |  |
| suh1 (F) | aataaagagactcgaagctgc | Sequencing *suh1* |
| suh1 (R) | CTATCAACGAAACTAGTTGGT |  |
| hot2 (F) | GCTGTAACCAGACGATATCAG | Sequencing *hot2* |
| hot2 (R) | CTTGACCACATGTGTACTCTC |  |
| prc1(F) | TGATAGGCACGATCGATACTC | Sequencing *prc1-1* |
| prc1(R) | CAGAGTTAATGTAGGACAACC |  |

**
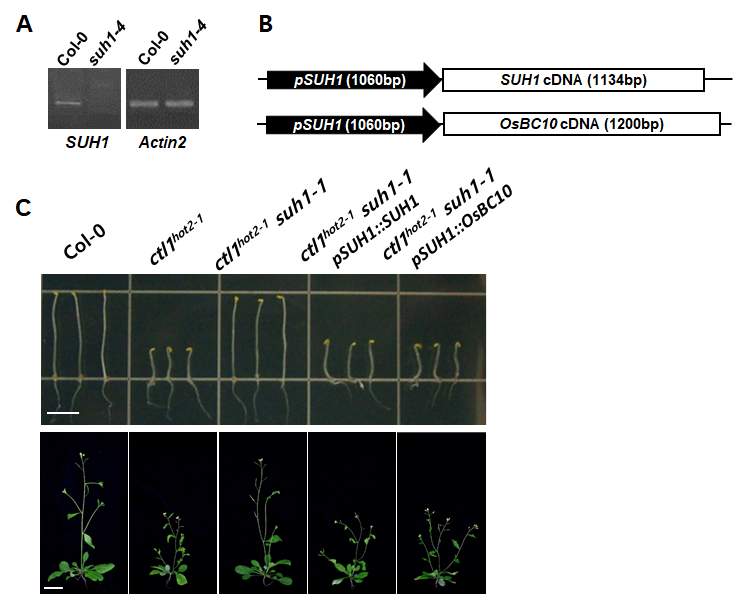
**

**Figure S1.** **Complementation of *ctl1^hot2-1^suh1-4* mutant plants.** (A) RT-PCR amplification of *SUH1* transcripts in wild-type and *suh1-4* mutant plants. *Actin2* was used as a loading control. (B) Schematic representation of the *pSUH1::SUH1* and *pSUH1::OsBC10* constructs used for complementation tests. The full-length cDNAs of *SUH1* and *BC10* were placed under the control of the *SUH1* promoter sequence (1,060 bp) in the binary vector pBI121. (C) Five-day-old dark-grown seedlings (top) and six-week-old mature plants (bottom) of Col-0, *ctl1^hot2-1^*, *ctl1^hot2-1^ suh1-4*, and two transgenic *ctl1^hot2-1^ suh1-4* plants carrying either the *pSUH1::SUH1* or *pSUH1::OsBC10* construct. The scale bars at the top and bottom represent 5 mm and 15 mm, respectively.

**
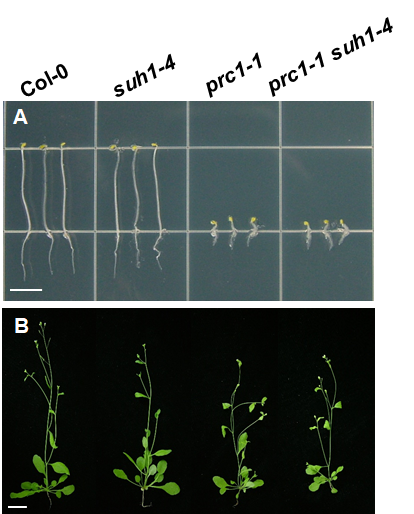
**

**Figure S2. Genetic interactions between *suh1-4* and *cesa6^prc1-1^*.** The *suh1-4* mutation does not restore the growth defect of *cesa6^prc1-1^*. (A) Five-day-old dark-grown seedlings. (B) Six-week-old light-grown plants of the genotypes Col-0, *suh1-4*, *cesa6^prc1-1^*, and *cesa6^prc1-1^ suh1-4*. Scale bars, 5 mm (A), 15 mm (B).

**
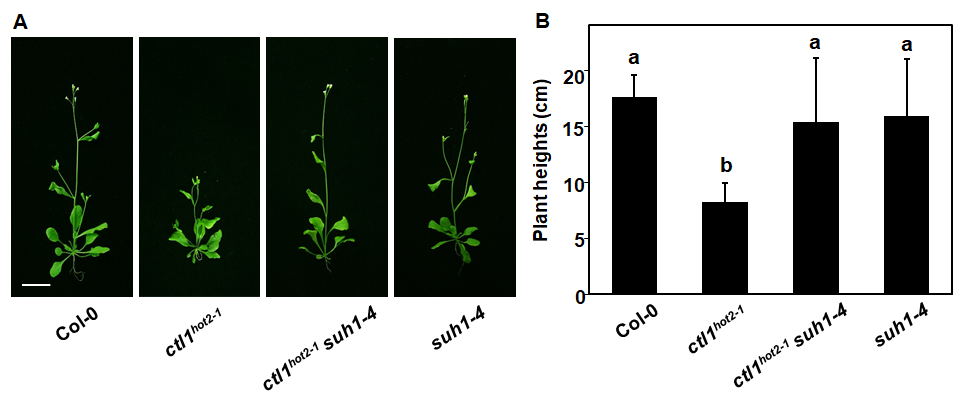
**

**Figure S3. Growth phenotypes of wild-type and mutant plants.** (A) Wild-type and mutant plants were grown in the soil inside a growth chamber (16 h light/8 h dark, 22 °C/18 °C cycle under a light density of 120 μmol m^-2^ s^-1^) for 6 weeks before being photographed. Scale bars, 15 mm. (B) Quantification of heights of wild-type and mutant plants described in (A). Data are presented as the mean ± *SE* of three replicates of 15 seedlings each. Statistically significant differences are indicated with different letters (one-way ANOVA followed by Tukey’s test, P < 0.05).

**
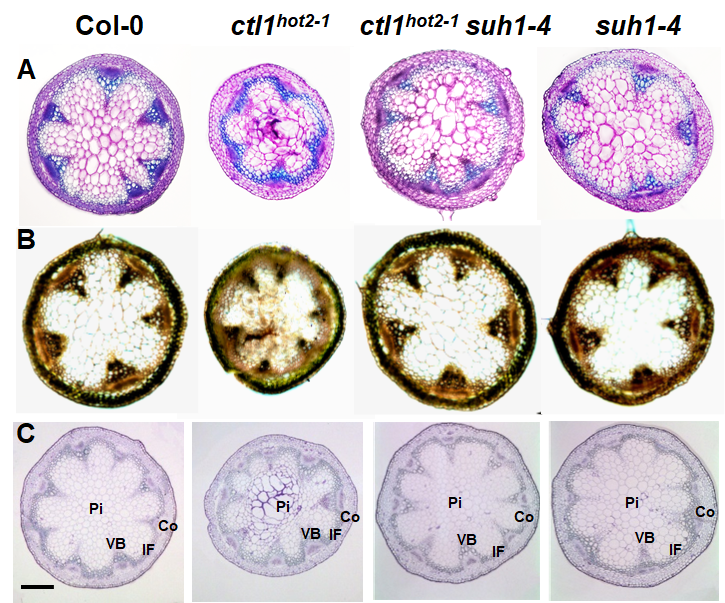
**

**Figure S4. Comparison of stem characteristics of wild-type and mutant plants.** (A, B) Hand-cut sections of the inflorescence stem of 6-week-old plants were stained with toluidine blue (A), or β-glucosyl Yariv (β-GlcY) reagent (B). Toluidine blue stains the cell wall containing lignin and pectin in blue-green and pink, respectively. β-GlcY stains arabinogalactan proteins (AGPs) in reddish brown. (C) Transverse sections of the inflorescence stems of 6-week-old wild-type and mutant plants were stained by toluidine blue. Pith (Pi), vascular bundle (VB), interfascicular fiber (IF), and cortex (Co) are visible. Sections were prepared at the first internode of the stem. Scale bar, 200 µm.

**
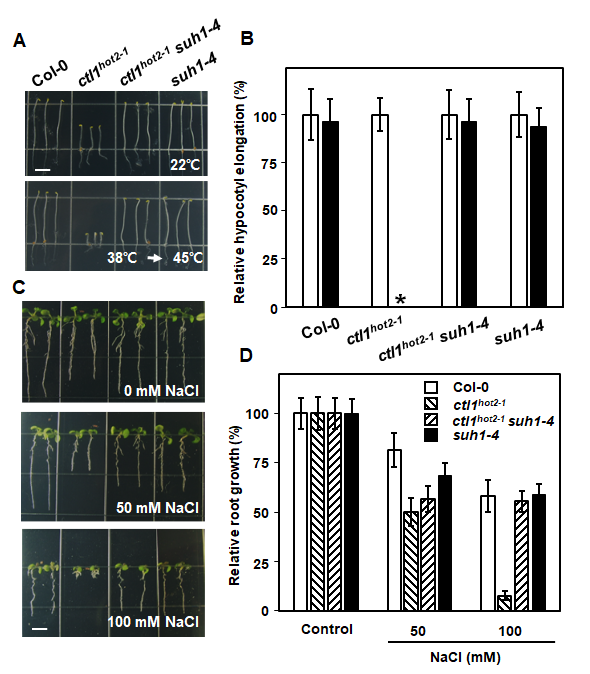
**

**Figure S5.** **Responses of wild-type and mutant plants to heat and salt stress.** (A) Thermotolerance assay of wild-type and mutant plants. Seedlings were grown in the dark at 2.5 days old and pre-treated at 38 °C for 90 min, then at 22 °C for 120 min. Seedlings were further stressed at 45 °C for 120 min and grown at 22 ℃ for 2.5 days before photographing. Seedlings grown in the dark for 5 days at 22 °C were used as controls. Scale bars, 5 mm. (B) Quantitative assessment of acquired thermotolerance of the seedlings shown in panel (A). The hypocotyl elongation levels in the seedlings subjected to the thermotolerance assay are expressed as a percentage of those in the seedlings of the same genotype grown for 5 days at 22 °C in the dark (controls). White and black bars indicate hypocotyl elongation under control and heat shock conditions, respectively. An asterisk indicates no elongation after heat shock, which was observed in *ctl1^hot2-1^* mutants. The mean and *SE* were calculated using data from three independent measurements, each with at least 15 seedlings. Scale bar, 1 cm. (C) Salt tolerance of wild-type and mutant plants as measured by root growth. Three-day-old seedlings grown vertically in half-strength MS medium were transferred to a medium containing indicated concentrations of NaCl and grown for 7 days. (D) A quantitative assessment of the salt sensitivity of the seedlings shown in panel (C). The mean and *SE* were calculated using data from three independent measurements, each with at least 15 seedlings. Root growth under saline conditions was expressed as a percentage of those grown under non-stress conditions.


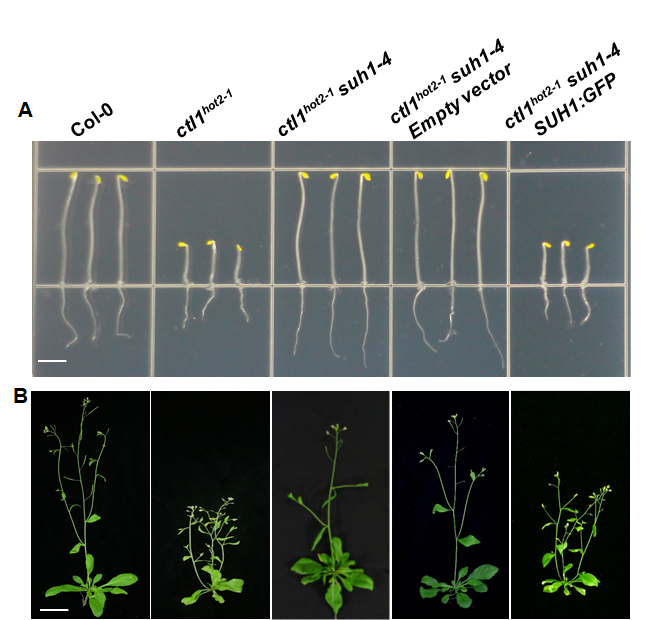


**Figure S6. Complementation of *ctl1^hot2-1^ suh1-4* plants by the introduction of the *SUH1-GFP* construct.** Five-day-old dark-grown seedlings (A) and six-week-old light-grown plants (B) of the genotypes Col-0, *ctl1^hot2-1^*, *ctl1^hot2-1^ suh1-4*, and two transgenic *ctl1^hot2-1^ suh1-4* plants carrying either empty vector and the *pSUH1::SUH1-GFP* construct. Scale bars, 5 mm (A), 15 mm (B).
