# Supplementary material for: Antagonistic functions of CTL1 and SUH1 mediate cell wall assembly in Arabidopsis
Source: Plant Direct. 2024 Mar 23;8(3):e580. doi: 10.1002/pld3.580 (PMC10960159; doi:10.1002/pld3.580)
Supplement: Supplementary file 1 — Data S1. Peer review [file PLD3-8-e580-s001.pdf]

Plant Direct
Open Access

Antagonistic functions of CTL1 and SUH1 mediate cell wall assembly in Arabidopsis

Nguyen Thi Thuy, Hyun-Jung Kim, Suk-Whan Hong*

Decision Letter Round 1:

December 20, 2023

Prof. Suk-Whan Hong
Chonnam National University
Gwangju
Korea (South), Republic of

RE: Antagonistic functions of CTL1 and SUH1 mediate cell wall assembly in Arabidopsis

Dear Dr. Hong:

Thank you for submitting to Plant Direct. All required reviews have been returned and we have now finished our evaluation of your manuscript. In light of the reviewers' and editor's comments, further revisions are needed before the paper can be accepted for publication in Plant Direct.

Please view the editors' and reviewers' comments below and use their suggestions as a guide while you work on your revision. In particular, authors are encouraged to expand the material and method section, provide high-resolution images, and tone down their strong statement or provide additional experiments/analysis. Reviewers also made constructive comments to improve the manuscript.

When uploading the revised version of this article, please be sure to include the following:

-A word document that contains your response to the reviewers. You should respond to each reviewer comment and note the changes made to the manuscript. If you do not agree with a reviewer's comment and choose not to make a suggested revision, please explain why. Please try to provide as complete an answer as possible to each reviewer's criticisms in the "response to reviewers" section.
-A tracked changes document
- A clean version of the latest version of the manuscript

To upload your revision, please click the link below.
https://plantdirect.msubmit.net/cgi-bin/main.plex?el=A2Lr2ew3A7oYg5I4A9ftdlgovfzT61uOqzJx7bDPgBwZ

In order to provide as timely a service as possible, we ask that your revision is resubmitted within three months after receipt of this request. If an extension is needed, please send a request, along with a brief explanation, to the editorial office at plantdirect@wiley.com .

Thank you very much for giving us an opportunity to review your work. I look forward to receiving the next version.

Sincerely,
Ying Gu

-- Reviewer comments:
Reviewer #1:

Thuy and co-authors report the identification of SUH1 mutations as suppressors of cell wall and growth defects in Arabidopsis ctl1 mutants.
The experiments that have been performed to characterize suh1 mutants clearly support all the statements made in this very well written manuscript. Results are described precisely and all figures are of high quality and in line with the main text. Materials and methods are well described and appropriate.

Specific comments:
1. Were only three suppressor mutants isolated from the screening, and all in SUH1? Or were additional suppressor mutations in other genes identified? This information would be very valuable for the interpretation of the results (P.11, lines 258-259).
2. THE1 is not directly participating in cellulose synthesis, but it's mutation can nevertheless rescue cellulose-dependent growth defects in Arabidopsis hypocotyls. The conclusion on page 12, line 289 should therefore be adjusted.
3. The exact mode of action of isoxaben is not known. The sentence on page 13, line 338, might be changed to e.g. "ISX is known to induce CESA internalization, while...".
4. The data supporting complementation of the ctl1 suh1 phenotype by SUH1-GFP should be shown in the supplement. This is important, since conclusions are drawn regarding the subcellular localization of SUH1 from investigation of this line (P. 15, line 372).
5. In the introduction, phenotypes of several CESA mutants are described without mentioning that the affected genes express CESAs. Please describe briefly the composition of cellulose synthesis complexes and mention that ixr1, ixr2, irx1 and irx3 are mutant alleles of CESA3, CESA6, CESA8 and CESA7, respectively. Similarly, qua1 and irx8 should be introduced as galacturonosyltransferases.
6. P. 4, line 82: Should read "glycoside hydrolases" instead of "glycosidic hydrolases".
7. In the discussion it is suggested that CTL1 and SUH1 "mediate" the same step in a multistep pathway. This might be misleading, since both gene products seem to have antagonistic functions (as

indicated already in the title). Instead, the authors could write that they "regulate" the same step.

Reviewer #2:

The project is very interesting, and the author has done a lot of works. However, there are several data that needs to be polished and additional analysis needs to be done in order to confidently conclude the role of SUH1 in relation to CTL1 and cell wall integrity.
1. It will be better if the author includes a brief explanation about hot2-1 in the introduction and not immediately jump into suppressor hot2-1 and ctl1hot2-1 seedlings.
2. Is there any particular reason for the author to choose Landsberg for the mapping?
3. It is important for the author to clarify the importance of looking at 2 different growth condition (dark-grown and light-grown), why the experiments were done with those 2 conditions? Also:
a. Please put the detailed growth condition in the materials and methods.
b. When the author put `light-grown` condition, how many hours of light/day is that?
4. Since the mutants are related to etiolation, then instead of using 6-weeks-old light-grown mutants (since it is not written, then I assume the author used a normal growth condition), isn`t it better to use short-day condition? Then, the author might still see the alteration in the phenotype and cell wall composition without extremely compromising the mutants´ growth and development.
5. For Figure 2:
a. Figure D-G need a better resolution.
b. Figure D and E seem overexposed. Are they overexposed? Then they might need to be re-adjusted.
c. Figure G has a little bit of blue hue to it. Did the author use some kind of staining there? If yes, please write it down.
6. Regarding suh1-4:
a. Although five-day-old dark-grown seedlings shows the same phenotype as wildtype, but in figure S2B, the inflorescence stem of light-grown suh1-4 seems shorter. Have the author measure this and check whether that difference is statistically significant or not. Depending on this measurement, then the statement in line 306 - "suh1 single mutation does not affect growth" might need to be revisited.
b. Line 302-305: Does the author have any hypothesis regarding what kind of process might happen to compensate the reduction in cellulose in suh1-4? Has the author look into other cell wall components to determine which one of them that might involve in the compensatory pathway?
7. Line 309-311: There is not enough evidence to conclude that lignin and pectin deposition in both ctl1hot2-1suh1-4 and suh 1-4 are indistinguishable from wildtype, since there`s no quantitative measurement and there`s not enough resolution in the image to show it. Based on my observation, ctl1hot2-1suh1-4 and suh 1-4 seem to have stronger staining for pectin. In addition, the blue-stained area in suh 1-4 is wider and more prominent than ctl1hot2-1suh1-4. I think, additional measurements are required to make that conclusion. There are several options that the author can choose to do:
a. Cell wall composition analysis (biochemistry).
b. Image analysis with imageJ or other imaging software by measuring the total area of pectin (only the pink-stained walls) divided by total root area (same with lignin too). The author also requires to put a better resolution of image in which the stained-walls can be clearly defined thus region-of-

interest can be selected.
c. Labelling with commercial antibodies against pectin (JIM probes) and check it with confocal microscope.
8. Line 318-322: the whole explanation regarding cell wall integrity threshold is not completely correct. My understanding when I was reading this that the author seems to suggest that the amount cellulose is directly determined the cell wall integrity threshold. So, when the cellulose content is above a certain limit, then it is above the cell wall integrity threshold then the phenotype is not affected. This statement is not correct.
Cell wall integrity maintenance is a continuous process involving all cell wall components, not only cellulose. This is why the experiment/measurement that I suggested in no.7 is important. Just because a mutant doesn`t show any growth phenotype, doesn`t mean that its cell wall integrity is not affected. There are a lot of compensatory pathway involves in cell wall integrity maintenance. For example: if a mutant with significantly reduced cellulose showing the same phenotype as wildtype, then there must be other pathway which is triggered to compensate the impaired cell wall integrity. Therefore, other cell wall components need to be checked. If that mutant then has more pectin, then it means impaired cell wall integrity (caused by reduced cellulose) triggers cell wall remodeling and boosts pectin deposition.
Seeing that suh1 managed to partially recover cellulose content, it will be interesting to see whether suh1 affects or triggers any compensatory pathway in cell wall remodeling during cell wall integrity maintenance.
9. For Figure 5: The author should include any cell wall or plasma membrane marker/dyes (such as calcofluor white or FM dyes) to better understand the localization in respect to the cell outline because I can see that the SUH1-GFP signal is not completely overlap. Therefore, the line 376 needs to be revisited. Whether it is a background noise or just due to a different focal point, but at this point it is difficult to judge because I can`t see the cell outline. However, if it isn`t possible to redo the imaging, then could the author provide the DIC image of that same confocal image of Figure 5B?
10. Line 372: Could the author provide the data (at least in the supplementary)?
11. Regarding the discussion:
There is not enough evidence on the cell wall`s perspective for the author to reach an undisputable conclusion regarding SUH1`s role in the regulation of cell wall integrity. Depends on the author`s response for point no. 6, 7, and 8 from this feedback, the discussion regarding CTL1`s and SUH1`s role might need to be revisited or rephrased. Maybe the use of less strong conclusion/statement is preferrable.
12. General conclusion:
Technically in cell wall research, we cannot just disregard the effect of our gene-of-interest towards other cell wall components. In order to make a confident claim that the gene-of-interest involves in specific cell wall biosynthesis (in this case is cellulose), then we have to check the status of the other wall`s component. Therefore, in the research involving cell walls, the author needs to at least have some experiments that can clearly show that the gene-of-interest only or mainly affects a specific cell wall component. The bare minimum is cell wall composition analysis or with proper microscopy techniques and image analysis.

Plant Direct

Open Access

Decision Letter Round 2:

February 29, 2024
Prof. Suk-Whan Hong
Chonnam National University
Gwangju
Korea (South), Republic of

MSID: 2023-01378-TWR1
MS TITLE: Antagonistic functions of CTL1 and SUH1 mediate cell wall assembly in Arabidopsis

Dear Dr. Suk-Whan Hong:

I am pleased to inform you that your manuscript "Antagonistic functions of CTL1 and SUH1 mediate cell wall assembly in Arabidopsis" has been accepted for publication in Plant Direct.

Your article will appear online in the next available issue of Plant Direct. To ensure your article gets published as quickly as possible, please pay attention to the steps detailed below. We have found that most of the delays happen at this stage, especially at the payment stage, so please respond as quickly as possible when prompted.

License Agreement: Once your article has been accepted it will move to Production and undergo admin and file checking - you may receive an email with any queries we have at this stage. When all required items are received by the publisher and queries resolved, the corresponding author will receive an email from Wiley's Author Services system which will ask them to log on at https://authorservices.wiley.com/bauthor and will present them with the appropriate license for completion. Your article cannot be published until both the signed license agreement and payment of the article fee have been received.

Payment of the Open Access Article Publication Fee: All articles published in Plant Direct are fully open access: immediately and freely available to read, download and share. Plant Direct charges a publication fee to cover the publication costs. The corresponding author for this manuscript should have already received a quote from the payments team (cs-openaccess@wiley.com) with the estimated article publication fee; please email cs-openaccess@wiley.com if this has not been received. The corresponding author should log on to the Wiley Author Services site, where the publication fee can be paid by credit card or an invoice. Pro Forma can also be requested. Payment of the publication charge must be received before the article will be published online.

Proofs: You will have the opportunity to look over your paper once more when you receive the author proofs for your article. The proofs will be with you in approximately two weeks. Please note that, in addition to publishing reviewer comments, the author's responses to review comments will also be published alongside the final version of the paper. If you would not like the author's responses to be published, please contact the editorial office at plantdirect@wiley.com .

Plant Direct
Open Access

Promotion of your article: You can help your research get the attention it deserves! Check out Wiley's free Promotion Guide for best-practice recommendations for promoting your work at www.wileyauthors.com/eeo/guide. And learn more about Wiley Editing Services which offers professional video, design, and writing services to create shareable video abstracts, infographics, conference posters, lay summaries, and research news stories for your research at www.wileyauthors.com/eeo/promotion.

Thank you again for your contribution to Plant Direct. If you have any questions, feel free to contact the editorial office at plantdirect@wiley.com .

Sincerely,

Ying Gu

Hsou-min Li

Editor, Plant Direct

--- Editor comments

--- Reviewer comments:
Reviewer #1:

Thuy et al. provide a very thorough revision of their manuscript on the antagonistic functions of CTL1 and SUH1. The Introduction was completely reorganized to address the concerns of both reviewers. Throughout the manuscript, the authors have revised the description of their results and toned down overstatements. Missing data was added and the description of methods amended where necessary.
The manuscript has been substantially improved by the revision and I have no further concerns.

Reviewer #2:

The author has addressed all feedbacks and concerns from the previous versions. The current version of the manuscript has been highly improved with a clearer objectives and discussion. All images also in much better resolutions and data that were presented here, can clearly support the author`s discussion and conclusion.

Author Response:

Plant Direct
Open Access

Dear Editor, I am sincerely grateful to the reviewers who took the time to provide excellent suggestions to improve the manuscript. This document contains point-by-point responses to their comments, which have been briefly summarized and arranged in order. Each comment has been written in bold font, with the reply provided in standard font. This manuscript aimed to isolate suppressor mutations (suh) that restore the multiple defects caused by a ctl1 mutation and to elucidate the interaction between CTL1 and SUH1. Overall, these findings should lead to an understanding of how suh1 suppresses ctl1-mediated defects in Arabidopsis. The function of SUH1 on cell wall assembly and integrity will be better investigated in more detail through our ongoing research. The statements highlighted by the reviewers as lacking sufficient evidence were revised based on the reviewers' comments to improve the manuscript. I am delighted that this revision better represents the objectives of my report. The specific responses to the reviewer's comments are as follows. Replies to Reviewer 1's comments. 1. The reason why three mutations belong to the same gene: Initially, three suppressor mutants were preferentially isolated and analyzed. However, it was not my understanding that they could belong to the same gene. Numerous resources have been applied to their research, meaning the isolation of more suppressor mutations could no longer be performed. Recently, two additional suppressor mutations have been isolated, although they have not been included in this paper because their mapping and allelism analysis were not presently conducted. 2. Line 289: Based on the reviewer's comment, the previous sentence was changed to "This implies that SUH1 is involved in cell wall assembly associated with CTL1 rather than directly responding to the lack of a functional CESA6 in Arabidopsis." (lines 302–303). 3. Line 338: Based on the reviewer's comment, the previous sentence was changed to "In addition, suh1-4 was more sensitive to DCB than to ISX, which induces CESA internalization. However, DCB affects microtubule-associated proteins (MAPs) that play a key role in vesicle transport." (lines 354–356). 4. Based on the reviewer's comment, the data supporting the complementation of the ctl1hot2-1 suh1-4 phenotype by SUH1-GFP construct were attached to Supplementary Fig. 6 (line 389). 5. Additional description of the characteristics of cell-wall-related mutations in the introduction: Based on the reviewer's comments, the composition of the cellulose synthesis complex and the protein characteristics of genes involved in cell wall synthesis were briefly described (lines 80–104). 6. Line 82: "glycosidic hydrolases" were replaced by "glycoside hydrolases" (line 50). 7. In the Discussion: Based on the reviewer's comment, "mediate" in the Discussion was changed to "regulate" (line 438, 447, and 464). Replies to Reviewer 2's comments 1. The introduction starts with a brief introduction of hot2-1: Based on the reviewer's comment, the introduction begins with a description of chitinase and hot2-1 to outline better the research goal of this manuscript (lines 50–68). 2. The particular reason for choosing the Lansberg ecotype for the mapping analysis: Molecular markers required for mapping are mostly published between the Columbia (Col-0) and Landsberg (Ler) ecotypes in Arabidopsis. Therefore, selecting Ler as the counterpart for mapping a mutation of hot2-1 suppressor in the Col-0 background is desirable. 3. Important reasons for looking at two different growth conditions (seedlings grown in the dark and light): CTL1 mutations inhibit hypocotyl elongation under dark conditions, which can be readily exploited to isolate their suppressors. Thus, demonstrating whether this recovery is inherited in successive generations is the key to confirming the isolation of suppressor mutations. Additionally, CTL1 mutations cause increased branching and semi-dwarfism under light conditions. Therefore, it is crucial to determine the effect of suppressor mutations on these defects. These processes and results are further described in my manuscript (lines 266–274). a. The detailed growth condition in the "Materials and Methods": The growth conditions for both the

dark and light conditions have been added to “the Materials and Methods section” (lines 117–122). b. Specification of light/dark cycle in growth chamber: The light/dark cycle in the growth chamber has also been described in “the Materials and Methods section” (lines 117–122). 4. Growth under short-day conditions: This research aimed to characterize suppressor mutations of ctl1hot2-1 mutation. Examining the effect of SUH1 mutation under the same conditions in which CTL1 mutation defects were confirmed in previous reports should be a priority. Therefore, the growth and development of suh1 mutants were investigated in this manuscript under the same long-day conditions rather than shortday conditions. 5. For Figure 2: a. Figure D-G need a better resolution: Based on reviewers' comments, Figure 2 has been modified to make the overall content simple and straightforward by increasing the resolution of the figures. First, the lignin staining of the stem has been replaced with a better resolution Figure of the seedlings grown in the dark (Fig. 2C). In addition, the results of pectin and AGP staining and stem cross sections have been transferred to the Supplementary Information (Supplementary Fig. S4). b. Figures D and E seem overexposed: As described above, the previous Fig. 2D has been removed and replaced with lignin staining of etiolated seedling (Fig. 2C). Additionally, the previous Fig. 2E was replaced with a less exposed and transferred to the Supplementary Information (Supplementary Fig. S4A). c. Figure G has a little blue hue: Fig. 2G represents the toluidine blue staining, as described in the “Materials and methods” section. When saving the images, a blue hue most likely resulted from the software process. No additional staining methods were used. 6. Regarding suh1-4: a. Line 306: The sentence on line 306 was removed entirely. Instead, it has been stated that the average height of the suh1-4 mutant appears to be slightly shorter than in the wild-type plants under optimal growth conditions, although there is no statistical difference (lines 313–315; Supplementary Fig. S3). b. Lines 302-305: Based on the reviewer’s comment, the statement presented in lines 302 to 305 was also deleted. I highlighted that suh1 mutation suppresses the multiple defects caused by ctl1hot2-1 mutations (lines 324–332) and that there are similarities in the defects between ctl1hot2-1 and suh1 (lines 334–340). This manuscript did not aim to investigate the effect of suh1 on altering the cell wall composition in Arabidopsis. The effects of suh1 on altering the cell wall components under various conditions will be better characterized in detail in future papers. 7. Lines 309-311: The previous statement on lines 309 to 311 was also deleted because the experimental results did not provide enough evidence to conclude that lignin and pectin deposition in ctl1hot2-1 suh1-4 and suh1-4 mutant plants were indistinguishable from those in wild-type plants. In addition, I highlighted that these histochemical analyses showed the recovery of ctl1hot2-1 -mediated defects by suh1 mutation but not the effect on their quantitative amount (lines 324–330). 8. Lines 318-322: I agree that the explanation regarding the cell wall integrity threshold described on lines 318–322 was incorrect. Therefore, the overall contents were reduced by removing the sentence (lines 318–322, old version). Instead, I described the similarity between the defects in the suh1 and ctl1hot2-1 mutant plants, albeit to different degrees and the recovery from ctl1hot2-1 -mediated defects following the suh1 mutation (lines 337–340). 9. For Figure 5: To confirm the cell outline easily, the DIC image of that same confocal image has been provided in Fig. 5B. 10. Line 372: Supplementary Fig. 6 shows the complementation of ctl1hot2-1 suh1-4 mutant plants by introducing the SUH1-GFP construct (line 389). 11. Regarding the Discussion: It is acknowledged that there is insufficient evidence to determine the role of SUH1 in cell wall integrity regulation. Therefore, most of the contents related to comments 6, 7, and 8 were replaced with descriptions of the isolation and characterization of suppressor mutations that restore multiple defects caused by ctl1 mutation (lines 308–340). In

addition, the aim of this report was reiterated at the start of discussion (lines 431–436). The statements that seemed to lack experimental evidence were deleted (lines 449–454, old version), and at the end, it has been noted that further studies are required to elucidate the possible roles of SUH1 in cell wall assembly (lines 475–479). 12. General conclusion: Following the reviewer's comments, all statements made without sufficient evidence to corroborate them were removed and replaced with descriptions of the isolation of suppressor mutations for ctl1 mutation and their characterization. Although it is important to study the roles of SUH1 on cell wall assembly, the priority of this manuscript was to attempt to understand the molecular mechanisms of suh1-mediated suppression of multiple defects caused by ctl1 mutation. At the end of the discussion, I also highlighted the need for further research to identify endogenous substrates of SUH1 and to study its roles in cell wall assembly.
